# OWL Reasoning: Subsumption Test Hardness and Modularity

**DOI:** 10.1007/s10817-017-9414-8

**Published:** 2017-06-08

**Authors:** Nicolas Matentzoglu, Bijan Parsia, Uli Sattler

**Affiliations:** Information Management Group, University of Manchester, Manchester, UK

**Keywords:** OWL, Ontologies, Reasoning, Modules, Subsumption testing

## Abstract

Reasoning with $\mathcal {SROIQ(D)}$, the logic that underpins the popular Web Ontology Language (OWL), has a high worst case complexity (N2Exptime). Decomposing the ontology into modules prior to classification, and then classifying the composites one-by-one, has been suggested as a way to mitigate this complexity in practice. Modular reasoning is currently motivated by the potential for reducing the hardness of subsumption tests, reducing the number of necessary subsumption tests and integrating efficient delegate reasoners. To date, we have only a limited idea of what we can expect from modularity as an optimisation technique. We present sound evidence that, while the impact of subsumption testing is significant only for a small number of ontologies across a popular collection of 330 ontologies (BioPortal), modularity has a generally positive effect on subsumption test hardness (2-fold mean reduction in our sample). More than 50% of the tests did not change in hardness at all, however, and we observed large differences across reasoners. We conclude (1) that, in general, optimisations targeting subsumption test hardness need to be well motivated because of their comparatively modest overall impact on classification time and (2) that employing modularity for optimisation should not be motivated by beneficial effects on subsumption test hardness alone.

## Introduction

Reasoning in popular, very expressive Description Logics (DL) is very difficult—deciding satisfiability in the Description Logic $\mathcal {SROIQ}$ for example is N2Exptime-complete [20]. Perhaps surprisingly, modern reasoning systems suitable for the entirety of OWL 2 DL (essentially a notational variant of $\mathcal {SROIQ}$) such as FaCT++ [38], Pellet [34], HermiT [9] and recently Konclude [37] often perform well against naturally occurring ontologies. However, there are still cases for which reasoning takes an unacceptable amount of time [21, 26], and the quest for optimisations is ongoing.

Locality-based modules are exploited to optimise Description Logic reasoning, both as a facilitator for incremental reasoning [13] and a means to optimise classification [31, 39]. Classification is the computation of subsumption hierarchies for classes and properties in an ontology. Intuitively, breaking the input problem (the ontology) into smaller pieces, reasoning over those pieces separately, then recombining the results, is appealing. There are a number of ways modularity can be be exploited for optimising classification: (1) Reducing the number of subsumption tests triggered during classification by reducing the search space; (2) Reducing the hardness of subsumption tests by getting rid of irrelevant axioms; (3) Integrating delegate reasoners to classify particular subsets of the ontology more efficiently; (4) Concurrent classification. To date, we have only a limited idea about what to expect from modularity as an optimisation technique:

- There has been, to our knowledge, no comprehensive investigation of how modularity effects reasoning, in particular to determine whether the effect is generally positive or negative, and then understanding the sources of those effects.
- The risk of hard subsets, i.e. subsets of an ontology that take longer to classify than the whole ontology, has been established [12]. However, while some speculations have been made that this risk does not apply to modules, there has been little solid evidence for this so far.
- There is a belief in the community that internal, non-module optimisations already localise enough to facilitate optimal subsumption testing, such that modularity would merely introduce overhead. This believe is partially grounded in the observation that, at least for ontologies that can be classified by state-of-the-art reasoners, most subsumption tests are easy.

If subsumption tests in a sub-module were generally easier than in a super-module (or the whole ontology), we could establish that, disregarding any overhead in computing the module, modularity would be a potentially viable optimisation technique. If, instead, a significant number of subsumption tests turned out to be harder in the sub-module, modularity would potentially pose a threat to optimisation. The goal of this paper is to determine whether subsumption test hardness can be systematically reduced using modularity alone and whether there are “pathological cases” where modularity could pose a threat to classification time. We conducted a comprehensive survey across BioPortal, a well known repository for biomedical ontologies, in order to isolate ontologies for which optimisations targeting subsumption test duration might be viable. We then continue to quantify the extent to which modularity-based optimisation was beneficial or detrimental to subsumption test duration, using a novel technique for sampling nested modules based on [12].

We find that the impact of subsumption test duration on overall classification time is significant only for a small number of ontologies, which generally threatens the effectiveness of optimisations that target subsumption test hardness *in general*. We further provide sound evidence that modularity generally has a positive effect on subsumption test duration: In our sample, which was not even concerned with generating the smallest possible modules, the subsumption test time was, on average (mean), reduced by a factor of 2. However, this positive effect was mainly due to a small proportion of tests which were significantly easier in the sub- than in the super-module, while the majority of the subsumption tests measured did not change at all (>52%), or only to a very small degree in hardness. Furthermore, the effects of modularity varied significantly between the reasoners. From this, we drive two major conclusions:

- New optimisations targeting subsumption test hardness need to be extremely well motivated, as subsumption test hardness does not play a major role when classifying most current ontologies. However, this only applies to ontologies that are already “cracked”; there might be room for optimising subsumption test hardness in more difficult ontologies that have not been successfully classified to date.
- Employing modularity for optimising classification performance should not be motivated by beneficial effects on subsumption test hardness alone. While we did observe some significant improvements in subsumption test duration on average, and a comparatively low number of pathological cases, the extent of this effect on overall classification time (14.2% mean reduction) appears insufficient to cover for the overhead of module extraction. On the positive side we learned that, at the very least, modularity is not detrimental to subsumption test hardness.

This work does not argue for or against using modularity for optimising classification *in general*. In fact, rather than optimal modules (the smallest possible modules required for a particular subsumption to be tested) we consider modules that comprise relatively large proportions of the ontology. Therefore, its is possible that the effect magnitude increases positively for reasoning techniques that employ very small modules. Furthermore, there have been a number of empirically successful cases where modular reasoning did make a significant difference by facilitating the integration of efficient delegate reasoners [31] or through divide and conquer [39]. Whether modularity is beneficial in other ways (traversal space reductions, parallelism) yet needs to be established.

## Background

We assume the reader to be familiar with the basic concepts of Description Logic and OWL. A **reasoner**, denoted $\mathcal {R}$ in the following, is a program that offers key logical services usually involving testing entailments in an ontology. The two most prominent families of reasoning algorithms for Description Logics are tableau-based and consequence-based. We are concerned with (hyper-)tableau^1^ algorithms, which are used for expressive languages beyond OWL 2 EL, QL or RL. For these (called the OWL 2 profiles), and others, specialised (and often consequence-based) reasoners exist. Logical services are, among others, classification, consistency checking, entailment checking, explanations and instance realisation. The reasoners under investigation in this paper are designed to implement key reasoning services for OWL, most importantly classification and consistency checking. Given an ontology (a set of axioms) $\mathcal {O}$, the signature of an ontology $\widetilde{O}$ is the set of named entities (classes, individuals, object properties, data properties) appearing in the axioms in $\mathcal {O}$. $\widetilde{\mathcal {O}}_{C}$ is the set of class names in $\mathcal {O}$, with $\widetilde{\mathcal {O}}_{C}\subseteq \widetilde{\mathcal {O}}$. Classification is the computation of subsumption hierarchies for classes, denoted $C(\mathcal {O},\mathcal {R},i)$, where $\mathcal {O}$ is the ontology being classified by reasoner $\mathcal {R}$ during a particular run of the reasoner *i*.^2^ More precisely, it is the process of determining whether $\mathcal {O}\models A\sqsubseteq B$ for any $A,B\in \widetilde{\mathcal {O}}_{C}\cup \{\top ,\bot \}$, thereby conducting a sequence of subsumption tests. We denote an individual subsumption test to determine whether $\mathcal {O}\models A\sqsubseteq B$ using reasoner $\mathcal {R}$ during reasoner run *i* as $ST(A,B,\mathcal {O},\mathcal {R},i)$. We use $time(C(\mathcal {O},\mathcal {R},i))$ (classification time) to denote the duration of the *classification* run *i* of reasoner $\mathcal {R}$ on $\mathcal {O}$, and, respectively, $time(ST(A,B,\mathcal {O},\mathcal {R},i))$ for the duration of a single subsumption test. The time of a task to finish is the difference of a timestamp recorded just after the task terminates with the timestamp taken just before the task started. A justification for an entailment $\alpha$ of $\mathcal {O}$ is a minimal set of axioms $\mathcal {J}\subseteq \mathcal {O}$ such that $\mathcal {J}\models \alpha$.

If it is clear from the context, we often omit the *time*() function symbol when talking about the time it takes to execute a particular task (such as classification or subsumption test time). Note that this notion of time does not say anything about how the time was spent, in particular does it not reflect anything like memory consumption or concurrency. It is a purely empirical notion that just says: given some way of obtaining a timestamp, how much time elapsed between the start and the end of the process.

Despite the staggering worst case complexity of deciding satisfiability, efficient algorithms have been developed for more than 20 years. The robustness [11] of current reasoners is largely a consequence of highly efficient optimisations. Some optimisations are tailored to particular ontologies [25], some apply only to a very specialised setting,^3^ some apply only to particular DL fragments and most only make sense in the context of a particular calculus. Classification typically involves a pre-processing phase and a traversal phase (which is intertwined with calls to the satisfiability engine). There are four types of optimisations that are of special interest to improving classification performance [3]:

1. *Pre-processing optimisations* try to rewrite the ontology in such a way that classification and subsumption testing becomes easier.
2. *Satisfiability optimisations* try to make satisfiability tests easier by optimising the satisfiability engine.
3. *Subsumption test optimisations* try to make tests faster by replacing them, either fully or partially, by cheaper ones.
4. *Traversal optimisations* are targeted at avoiding subsumption tests, for example by exploiting the typical tree-shape of the class hierarchy.

Three well known members of the family of *pre-processing optimisations* that are usually employed by traversal and tableau-based approaches are *normalisation*, *simplification* and *absorption* [16, 38]. Normalisation for example rewrites axioms in such a way that the satisfiability engine can detect clashes early on. This is particularly important because we can describe the same thing in different ways, for example $A\sqcap B$ and $B\sqcap A$, as well as $\lnot (A\sqcap B)$ and $\lnot A \sqcup \lnot B$. Absorption is an optimisation that attempts to reduce the high degree of non-determinism induced by general concept inclusions, for example by rewriting axioms of the for $A\sqcap B\sqsubseteq C$ to an axiom with an atomic left hand side: $A\sqsubseteq C\sqcup \lnot B$. One of the most important optimisations that is aimed at *making tests easier by optimising the satisfiability engine* is back-jumping. When exploring potentially deeply nested non-deterministic branches in a tableau setting, the algorithm generally retracts to the last non-deterministic choice and continues with the next branch. It is easily possible that the clash was caused by an interaction that was independent of the expression that caused the non-deterministic choice, which may result in it happening again. Back-jumping aims at recognizing such situations and enables retracting to a non-deterministic choice that would avoid the same clash happening again [36]. Among the optimisations for *making tests easier by replacing them with cheaper tests* is *caching of partial expansion trees*. This optimisation addresses the issue that many (potentially costly) satisfiability checks are repeated with minor variations (as part of other satisfiability tests). Cached partial expansion trees can be used to prove non-subsumptions without actually performing them. Other optimisations exploit graph properties to replace certain tests by look-ups to the transitive closure of the known subsumptions [3, 4, 16].

Given an ontology $\mathcal {O}$, a reasoner $\mathcal {R}$, a set of pairs $pairs(\mathcal {O})\subseteq \widetilde{\mathcal {O}}_{C}\cup \{\top ,\bot \} \times \widetilde{\mathcal {O}}_{C}\cup \{\top ,\bot \}$ and a classification $C(\mathcal {O},\mathcal {R},i)$. We use $trav(\mathcal {O},\mathcal {R},i)$ to denote the sequence of subsumption tests $(ST(A_i,B_i,\mathcal {O},\mathcal {R},i))_j$ that $\mathcal {R}$ carries out during $C(\mathcal {O},\mathcal {R},i)$ with ($A_i,B_i)\in pairs(\mathcal {O})$. The main sub-category of optimisations that aim at reducing the traversal space are traversal algorithms. The most important traversal algorithms are those based on the *Enhanced Traversal* [5] (ET), and more recently the *Novel Approach* [8] (NA). Another important technique to avoid unnecessary subsumption tests by exploiting the asserted knowledge are *told subsumers* [38].

Since $ST(A,\bot ,\mathcal {O},\mathcal {R},i)$ is regarded here as a subsumption test, we do not distinguish between subsumption and satisfiability checks; in that sense, “subsubumption test hardness” should be read as “subsumption test or satisfiability check hardness”. The same goes for ontology consistency, which we regard simply as the subsumption test $ST(\top ,\bot ,\mathcal {O},\mathcal {R},i)$.

A naive traversal algorithm would iterate through all ordered pairs of concept names $A,B\in \widetilde{\mathcal {O}}_{C}$ and determine whether $\mathcal {O}\models A\sqsubseteq B$, requiring a total of $n^2$ tests, where $n=|\widetilde{\mathcal {O}}_{C}|$. This is clearly inefficient, given the tree like structure of a typical class hierarchy. For example, if we find that $\mathcal {O}\not \models A\sqsubseteq B$ then we do not need to check whether $\mathcal {O}\models A\sqsubseteq D$ for any *D* with $\mathcal {O}\models D\sqsubseteq B$. Therefore, folklore suggests to assume a tighter upper bound of $n*log(n)$ tests, which reflects the complexity of traversing a binary tree. While this is (as we will see later) much closer to the empirical upper bound than $n^2$, $n*log(n)$ is occasionally exceeded in non-tree-like class hierarchies with multiple inheritance or shallow ones, for example if we have only one super class with a flat list of children.

### Using Modules for Reasoning

We are interested in determining whether modules can be effectively utilised to (1) make subsumption tests easier and (2) prune the traversal space. The particular flavour of “logically respectable” **modules** we use is based on *syntactic locality* [14]. Current modular classification approaches use so-called $\bot$-modules (bottom-modules) which have a number of desirable properties: (1) $\bot$-mod$(\varSigma ,\mathcal {O})\subseteq \mathcal {O}$ and (2) for a module $\mathcal {M}$ and an entailment $\alpha$ with $\widetilde{\alpha }\subseteq \widetilde{\mathcal {M}}$, $\mathcal {O}\models \alpha$ iff $\mathcal {M}\models \alpha$. (3) Furthermore, being based on syntactic locality, they can be extracted in polynomial time while being reasonably compact and exact [14]. Thus, $\bot$-modules are *classification complete* for their signature with respect to their parent ontology. Hereafter, we will use $\mathcal {M}$ to refer to a syntactic locality-based $\bot$-module. Other kinds of locality-based modules are available, such as syntactic $\top$-modules or semantic $\emptyset$-modules, which come with the same guarantees (1) and (2); guarantee (3) only applies to syntactic locality-based modules. The differences between these modules are of no consequence to this work. The interested reader may refer to [14].

Very recently, reasoner developers have started to **utilise modularity for classification**. They either are (1) using modules for incremental reasoning [13] or (2) using modules to improve classification time, like MORe [31] and Chainsaw [39]. In practice however, modular reasoning techniques do not always improve the performance of classification [10]. In fact, they can drastically impair performance, making it a hit and miss game to choose between a modular reasoner (e.g. MORe-HermiT, Chainsaw-JFact) and its monolithic counterpart (e.g. HermiT, JFact). Cases where modular techniques fail to provide any benefit can often be due to various kinds of overhead induced by modular reasoners (e.g. extracting the modules might take longer than classifying the whole ontology) or redundancy introduced by the mostly unavoidable and often significant overlap between the various modules extracted. In a set of experiments leading up to this work we observed a curious effect: Not only are there cases where there are individual subsumption tests that can be, often significantly, harder in a module extracted by a modular reasoner than in the whole ontology, and we could even observe that there are occasionally modules whose classification time exceeds that of the entire ontology $\mathcal {O}$ it was extracted from [24].

## Related Work

Attempts to understand DL reasoning performance are, up until today, rarely systematic or comprehensive. Since 2013, the ORE reasoner competition tries to establish methodological foundations for more reliable comparisons [10, 29] between different reasoners and across a range of different reasoning services. OWL Reasoner benchmarks are systematic empirical comparisons of reasoner performance against well defined ontology corpora. They have been conducted for varying purposes, for example (and most prominently) guiding end-users for selecting appropriate reasoners for their problem [6, 10] or understanding reasoning or the state of reasoning in general [11]. Dentler et al. [6] conduct a principled investigation to identify suitable criteria for choosing an appropriate reasoner for EL ontologies. In our work, we are interested in mapping out subsumption test hardness during full classification across reasoner-ontology pairs (phenomenological characterisation) and the potential of modularity to pinpoint counter-intuitive cases (i.e. harder tests in a sub-module). Another, relatively recent, branch of related work attempts to understand empirical reasoner behaviour and reasoning hardness through Machine Learning based prediction of reasoning time using ontology features [1, 2, 18, 19, 33].

Most benchmarks conduct an only semi-principled dataset selection: Even carefully executed benchmarks such as Dentler et al. [6] usually cherry pick a set of *somehow* relevant ontologies. Few works sample from existing corpora or the web, and only Gonçalves et al. [11], to the best of our knowledge, deal with corpora larger than 500 ontologies for independent reasoner benchmarking. In practice, the current de facto gold-standard corpus for ontology experimentation is BioPortal [28], which also provides a well designed infrastructure to obtain an interesting range of biomedical ontologies programatically. We are using a snapshot of BioPortal in this work. As far as we know, no benchmark to date has investigated *subsumption testing during classification* across reasoners in a principled manner. However, various benchmarks have investigated the effect of certain optimisations on subsumption test avoidance [5, 8]. While the literature on classification optimisation and reasoning is vast [16, 30, 35], little progress has been made in understanding classification hardness of real ontologies, both empirically and formally, with some notable exceptions [5, 8, 17]. In particular, there is no clear picture of the effect of subsumption test hardness on state-of-the-art corpora such as BioPortal [28].

## Reducing Subsumption Test Hardness Using Modules

In this paper, the phenomenon under investigation is **subsumption test hardness in the context of classification**. In the following, unless stated otherwise, a reasoner is a traversal/(hyper-) tableau style reasoner. A **subsumption test** occurs in $C(\mathcal {O},\mathcal {R},i)$ when the reasoner attempts to determine whether $\mathcal {O}\models A\sqsubseteq B$ by means of the underlying calculus. The **subsumption test hardness** characterises the time it takes to perform a subsumption test. For the purpose of this paper, the answer of a test is either yes or no. Note however that, for any given implementation, (1) more than just a binary answer may be computed and provided (e.g., pseudo-models [15] may be constructed and cached) and (2) no guarantee is given that the answer is correct (i.e. the reasoner might be buggy). **“In the context of classification”** means that we are not exploring individual “cold” tests, i.e. letting the reasoner decide whether $\mathcal {O}\models A\sqsubseteq B$ for some *A*, *B* from outside the classification process, because we want to understand the contribution of subsumption testing to classification as a whole, with all the optimisations involved. Note that we only measure actual subsumption tests, *omitting* most importantly the *initial consistency check*, which usually involves a call to the tableau engine. This is very important when interpreting subsequent results, especially the numbers of ontologies for which no subsumption test was recorded. It is also possible that the model generated during the consistency test is used to derive known (non-)subsumptions and disjointness axioms.

It is known that a random subset of an ontology $\mathcal {O}$ can be *pathological*, i.e. harder to classify than the whole ontology $\mathcal {O}$ [12]. One obvious example for this is missing a disjointness axiom high up the hierarchy between two concepts *A* and *B* (in the subset) that makes testing the mutual subsumption of all children of *A* and *B* obsolete. Given the existence of such pathologically hard subsets, it is not immediate that module-based reasoning is going to be a straightforward, much less useful, optimisation. In this section we give an analytical argument for the use of modules in classification.

An *experiment run* is a single execution of a program (for example a reasoning task of $\mathcal {R}$ over $\mathcal {O}$) on a defined experiment machine (typically a server node or desktop computer). We now define subsumption test hardness in terms of an aggregation function^4^ as follows: Given an ontology $\mathcal {O}$, a reasoner $\mathcal {R}$, two concept names *A* and *B*, an experiment run *i*, we call *individual subsumption test hardness* the duration $time(ST(A,B,\mathcal {O},\mathcal {R},i))$ it takes $\mathcal {R}$ to decide whether $\mathcal {O}\models A\sqsubseteq B$ in run *i*. Given a set of experiment runs *I* and an aggregation function $\varphi$, we call *subsumption test hardness* the time aggregated across *I* by aggregation function $\varphi$.

In other words, the hardness of a subsumption test between the concept names *A* and *B* is the time it takes to compute the subsumption in the context of an ontology $\mathcal {O}$, as computed by a reasoner $\mathcal {R}$, aggregated across different runs by the aggregation function $\varphi$. Please note that the run *i* of $\mathcal {R}$ takes place on an experimental machine, which we do not further specify, but we assume that it is implicitly encoded in *i*. For brevity, we always omit $\varphi$ because it should be either clear from the context (experimental design description) or irrelevant for the argument at hand; we also sometimes omit $\mathcal {R}$, if it is clear from the context (or irrelevant). Irrelevant in this context means that the argument should hold for any *fixed* $\varphi$ (or $\mathcal {R}$).

### Why Modular Reasoning Should Make Subsumption Testing Easier

Given a subsumption test $ST(A,B,\mathcal {O},\mathcal {R},\cdot )$, it should be the case that, for every two modules $\mathcal {M}_1\subset \mathcal {M}_2\subseteq \mathcal {O}$ with ${A,B}\in \widetilde{\mathcal {M}_1}$, the hardness of $ST(A,B,\mathcal {O},\mathcal {R},\cdot )$ and that of the $ST(A,B,\mathcal {M}_i,\mathcal {R},\cdot )$ is equal if we ignore the overhead involved in determining (ir)relevant axioms.^5^

The reasons for this are the properties of locality-based modules: not only is every justification for an entailment part of every module for the signature of that entailment, but any module $\mathcal {M}$ with $\mathcal {M}_{sub}\subset \mathcal {M}\subseteq \mathcal {O}$ is a model-conservative extension of $\mathcal {M}_{sub}$. As a consequence, the space of possible models for $\mathcal {M}$ is more complex than that for $\mathcal {M}_{sub}$ in two ways: (1) since every model for $\mathcal {M}_{sub}$ can be extended to a model of $\mathcal {M}$, the models for $\mathcal {M}$ are larger^6^ and (2) since models from $\mathcal {M}_{sub}$ could potentially be extended in multiple ways to models for $\mathcal {M}$, i.e., there is a 1-to-many relationship between models of $\mathcal {M}_{sub}$ and those of $\mathcal {M}$, the models for $\mathcal {M}$ are more numerous.^7^ Thus, the task of finding a model for $A\sqcap \lnot B$ or verifying that there are no models is, in principle, *at least as difficult* for $\mathcal {M}$ than for $\mathcal {M}_{sub}$ due to the larger space. An immediate consequence of the relationship between models of $\mathcal {M}$ and $\mathcal {M}_{sub}$ is that it cannot be the case that $\mathcal {M}_{sub}$ is consistent, while $\mathcal {M}$ is not (i.e. $\mathcal {M}$ has no models).

Let us consider the *case of a subsumption* where $\mathcal {M}_{sub}\models A\sqsubseteq B$, with $A,B\in \widetilde{\mathcal {M}_{sub}}$, i.e. $A\sqcap \lnot B$ is not satisfiable w.r.t. $\mathcal {M}_{sub}$. We call $\mathcal {J}_{\mathcal {M}}^{A\sqsubseteq B}$ the set of all justifications for $A\sqsubseteq B$ in $\mathcal {M}$. Module properties ensure that $\mathcal {J}_{\mathcal {M}_{sub}}^{A\sqsubseteq B}=\mathcal {J}_{\mathcal {M}}^{A\sqsubseteq B}$, hence for all ${\mathcal {J}_{i}}\subseteq \mathcal {M}$, if $\mathcal {J}_{i}$ is a justification for $A\sqsubseteq B$ over $\mathcal {M}$ then $\mathcal {J}_{i}\subseteq \mathcal {M}_{sub}$, see above. Since all justifications are available in both modules, reasoning should not be harder in $\mathcal {M}_{sub}$: there can be no easier reason for $A\sqsubseteq B$ in $\mathcal {M}$ than in $\mathcal {M}_{sub}$.

Let us now consider the *case for a non-subsumption* where $\mathcal {M}_{sub}\not \models A\sqsubseteq B$ ($A,B\in \widetilde{\mathcal {M}_{sub}}, A\sqcap \lnot B$ is satisfiable w.r.t. $\mathcal {M}_{sub}$). We call a model $\mathcal {I}$ of $\mathcal {M}$ with $(A\sqcap \lnot B)^{\mathcal {I}}\ne \varnothing$ a *counter-model* of $\mathcal {M}$ and $A\sqsubseteq B$. Let $\mathcal {I}\models \mathcal {M}_{sub}$ with $e\in (A\sqcap \lnot B)^{\mathcal {I}}$ be a counter-model for the subsumption in $\mathcal {M}_{sub}$. From the considerations above we have that counter-models for $A\sqsubseteq B$ over $\mathcal {M}$ may not be less numerous or smaller than those over $\mathcal {M}_{sub}$. In other words, every counter-model in $\mathcal {M}$ has a smaller or equal-sized counter-model in $\mathcal {M}_{sub}$. If we consider the variants of locality-based modules, this consequence may be slightly more restricted. For example, consider the following example ontology $\mathcal {O}_{exp}$:

1. $$\alpha _1: A\sqcup X_1\sqsubseteq X_2$$
2. $$\alpha _2: X_3\sqsubseteq A \sqcup X_4$$
3. $$\alpha _3: A\sqsubseteq \exists R.\top $$

The $\top$-module of (signature) $\varSigma =\{X_3,X_4,A\}$ is just $\mathcal {M}_{sub}=\{\alpha _2\}$. A counter-model for $A\sqsubseteq \bot$ would merely consist of a single individual which is an instance of A. If we tested the same entailment with respect to $\mathcal {O}_{exp}$, we would at the very least add a non-empty interpretation of *R*, so the counter-model for $A\sqsubseteq \bot$ in $\mathcal {O}_{exp}$ is bigger than in $\mathcal {M}_{sub}$. The same does not hold for $\bot$-modules. Given a module $\mathcal {M}$ and its parent ontology $\mathcal {O}$ and the terms outside the module $\varSigma _{x}=\widetilde{\mathcal {O}}\setminus \widetilde{\mathcal {M}}$, then every $\alpha \in \mathcal {O}\setminus \mathcal {M}$ “is” a tautology when terms in $\varSigma _{x}$ are replaced with $\bot$ ($\bot$-locality). As a consequence, if $\mathcal {I}$ is a model of $\mathcal {M}$, the “empty” extension of $\mathcal {I}$ to terms in $\varSigma _{x}$ ($\mathcal {I}'$) is a model of $\mathcal {O}$. However, building a tableau differs from the abstract notion of models. Highly optimised reasoners such as FaCT++ that do not know the modular structure of an ontology, sometimes generate models beyond the size strictly necessary. This can happen for example when dealing with general concept inclusions, which in turn depends on the many possible ways absorption is applied.

Note that the analytical argument presented in this section applies to tableau and hyper-tableau based classification algorithms and ignores the fact that many modern reasoners are hybrids that integrate multiple reasoning procedures based on different calculi.^8^ That means that axioms irrelevant for a particular subsumption to hold *can* make a difference in practice. For example, the removal of an inverse role that is irrelevant for determining the subsumption relation of two concepts enables the reasoner to resort to a cheaper reasoning procedure, therefore making the subsumption test potentially easier. Furthermore the *no-easier-justification* argument is rather theoretical: In practice, stochastic effects (both caused by algorithmic non-determinism and implementational aspects) can lead the algorithm into a harder space even when classifiying the sub-module.

### Empirical Definitions

Given a reasoner $\mathcal {R}$, two modules $\mathcal {M}_1\subset \mathcal {M}_2\subseteq \mathcal {O}$ and ${A,B} \in \widetilde{\mathcal {M}}_1$. Consider the possibilities:

1. $ST(A,B,\mathcal {M}_1,\mathcal {R},i) \in trav(\mathcal {M}_1,\mathcal {R},i)$, $ST(A,B,\mathcal {M}_2,\mathcal {R},j) \not \in trav(\mathcal {M}_2,\mathcal {R},j)$: In this case, an additional test was triggered in the sub-module.
2. $ST(A,B,\mathcal {M}_2,\mathcal {R},j) \in trav(\mathcal {M}_2,\mathcal {R},j)$, $ST(A,B,\mathcal {M}_1,\mathcal {R},i) \not \in trav(\mathcal {M}_1,\mathcal {R},i)$: A particular subsumption test was avoided altogether in the sub-module.
3. $time(ST(A,B,\mathcal {M}_1,\mathcal {R},i)) < time(ST(A,B,\mathcal {M}_2,\mathcal {R},j))$: This is the *expected* case–if we add axioms, the search space gets more complex.
4. $time(ST(A,B,\mathcal {M}_1,\mathcal {R},i)) \approx time(ST(A,B,\mathcal {M}_2,\mathcal {R},j))$: This case is reasonable if the implementation can (cheaply) recognise that the problem $\mathcal {M}_2\models A\sqsubseteq B$ can be restricted to $\mathcal {M}_1\models A\sqsubseteq B$. A naive modular reasoner can potentially achieve this simply by extracting $\mathcal {M}_1$ from $\mathcal {M}_2$ and reasoning over $\mathcal {M}_1$. Note that this might hurt overall classification time as we add in extraction overhead. We call this case *optimal*.
5. $time(ST(A,B,\mathcal {M}_1,\mathcal {R},i)) > time(ST(A,B,\mathcal {M}_2,\mathcal {R},j))$: This case is *pathological* as the, in principle, harder (or equally hard) problem turned out to be easier. Somehow, the extra information makes the reasoner do better in spite of being strictly irrelevant to the problem at hand.

The first two cases can be due to traversal non-determinism and subsumption avoidance and will not be discussed here; an in-depth analysis of these can be found in [23]. A possible explanation of the pathological case is that the implemented calculi are inherently non-deterministic; different choices can produce wildly different behaviour, and implementations make choices based on fallible heuristics. If the heuristics are sensitive to irrelevant information, then the effect of that irrelevant information might be to induce a significantly better choice by luck. Consider testing the satisfiability of a disjunction $C\sqcup D$, and that it is satisfiable because *D* is satisfiable and C is not. Obviously, we will typically do worse if we choose to explore *C* before *D* as determining the unsatisfiability of *D* would not be necessary. Suppose our disjunction selection heuristic is the length of the sub-expression and $|C|<|D|$. This is ok, and $time(SAT(C\sqcup D)) \approx time(SAT(C))$. Now, suppose we add a bit of information to *C* to get a $C'$ such that $|C|<|C'|<|D|$. Now $time(SAT(C'\sqcup D)) \approx time(SAT(C'))$. We are a bit worse off, but as expected. Now, suppose that $|D|<|C|$. $time(SAT(C\sqcup D)) \approx time(SAT(D))+time(SAT(C))$. If $time(SAT(D))\gg time(SAT(C))$ our heuristic made a detrimental choice. But suppose we extend *D* to $D'$ such that $|C|<|D'|$ and the information added was completely irrelevant to *C*. Now, even though $time(SAT(D))<time(SAT(D'))$, $time(SAT(C\sqcup D'))\approx time(SAT(C))$. That way, we can see how irrelevant information can interact beneficially with a heuristic.^9^

Moreover, depending on the source of the stochasticity,^10^ we might have highly variable time between runs of the very same module/reasoner pair. Consider for example the above optimisation, but instead of using sub-expression length as the heuristic, we replace it with random selection, or selection based on hash set traversal (no lexical order). In this case, we would, in some runs, get low values for $time(SAT(C\sqcup D))$, and, in other runs, high ones. Thus, any pathological case might merely occur because *in that run* $\mathcal {R}$ was unlucky with respect to $\mathcal {M}_1$ and/or lucky with respect to $\mathcal {M}_2$. This potential lack of stability induced by stochasticity (rather than “mere” measurement error) makes it difficult to explain module-varying behaviour.

In this study, we investigate two research questions as described in the following section. Across both studies, we say an ontology is *profiled*, if it falls under one of the 3 polynomial OWL 2 profiles (EL, QL, RL); we say an ontology is *pure DL* if it falls under the OWL 2 DL profile, but is *not* profiled; we say it is *full* if it is expressed in OWL 2 full, but is neither pure DL nor profiled. We group ontologies by size bins. Ontologies with less than one axiom are considered empty, with 1–9 axioms *very small*, with 10–99 axioms *small*, 100–999 *medium*, 1000–9999 *large*, 10,000–100,000 *very large* and with more than 100,000 logical axioms *huge*. These bins do not aim to reflect the actual distribution of ontology sizes. The main purpose of introducing them is to make some parts of the analysis easier to understand; therefore we chose the bin ranges in a way that is easily memorable by the reader.

### Research Questions and Metrics

The first research question aims at determining the relevance of subsumption testing for classification in general: *What is the relationship between subsumption test hardness and ontology classification time in practice (RQ1)?* How much time does a reasoner spend on “real” reasoning, compared to preprocessing and traversal? If subsumption testing is significant to an ontology’s classification for some reasoner, how is the time distributed across tests? Are there a few “killer” tests or do numerous easy tests dominate? Is difficulty randomly distributed across positive and negative tests? How many tests are done, and how effective are reasoners at avoiding tests? How much do the answers to these questions depend on the particular implementations?

RQ1 is important to understanding reasoning in general and in the design of modularity oriented procedures. For example, if it is typically the case that the total time spent on subsumption tests constitutes only a small fraction of the overall classification time, the importance of modularity for making tests easier or avoiding them is diminished (but there might still be other beneficial effects).

In order to judge the impact of subsumption tests on classification performance, we draw on three different metrics.

- Overall classification time (OCT): This is the overall time it takes the reasoner to perform all stages, from pre- to post-processing (see Sect. 5.3).^11^ This measure constitutes the upper bound for any gain through modularity. A very low absolute value may indicate that there is no need and space to further optimise.
- Sum of subsumption tests (SST): the sum of all times of subsumption tests triggered during a single classification run.
- Hardest subsumption test (HST): the duration of the hardest test triggered during a single classification.
- Sum of subsumption tests to overall classification time ratio (SST/OCT): This tells us something about how much time the reasoner spends in the context of the tableau engine. A large value (close to 1) can suggest a need for optimising test avoidance as well as for finding more efficient ways to determine subsumption. Another possibility is a skewed ontology, namely one with a very small signature and a comparatively large size. A low value (close to zero) renders attempts to improve the performance of the tableau engine irrelevant. Note that by itself, this number does not directly imply the potential applicability of modular techniques because modular techniques can in principle be beneficial in more ways than test avoidance or easyfication (for example by facilitating concurrent classification, or perhaps more efficient consequence-based reasoning).
  We say that subsumption testing has a *strong impact* on classification time if it accounts for more than 40% of the OCT. A medium impact is defined between 20 and 40% and a small impact between 0 and 20%.^12^
- Hardest subsumption test to overall classification time ratio (HST/OCT): This tells us something about the complexity of the reasoning problems inside an ontology. A large value (close to 1) means that a single test dominates the entire classification time. This raises the question whether it can be made easier or even be avoided altogether using modular techniques.
- Subsumption test count (STC): The number of subsumption tests triggered during classification. This number can be used to estimate the effectiveness of “normal” traversal algorithms (by comparing them against the $n^2$ and $n*log(n)$ upper bounds). Very low counts indicate that modularity might not be effectively usable to avoid further tests.

The second research question is of central importance for modularity-based classification: *How is a reasoner’s subsumption test performance sensitive to modularly irrelevant axioms? In other words, is the behaviour of current reasoners expected, optimal, or pathological (RQ2)?* If the behaviour is typically *expected* and we are dealing with ontologies for which subsumption testing has a strong impact, then there is a clear opportunity for explicitly module sensitive procedures and optimisations. A key sub-question here is whether the variance of reasoner performance between runs is sufficient to distinguish between stochastic performance variability and *module sensitive* performance variability. This is important in order to judge how reliably we can trace a single subsumption test through different sub-modules of an ontology, and may also give a warning sign for non-determinism, for example in the case that a test appears or disappears given a particular ontology-reasoner pair across runs. We will address the problem of measurement stability mainly by (1) looking at the *coefficient of variation* (COV) of subsumption test hardness, across different runs and (2) isolating cases where the classification time was potentially influenced by (obvious) stochastic effects. We use *varying number of tests triggered* across multiple runs as a first lower bound to label ontology-reasoners pairs as influenced by stochastic effects.

## Experimental Design

We have conducted two separate experiments, each addressing one of our two research questions: (1) The characterisation of subsumption test hardness in the context of classification across BioPortal, addressing RQ1; (2) the in-depth analysis of a subset of BioPortal for exploring the effect of modularity on subsumption test hardness, addressing RQ2.

We conducted our study on a snapshot of the BioPortal ontology repository of January 2015 (330 non-empty ontologies), which was extensively described in [23]. In terms of infrastructure, we used a set of four (equivalent) Mac Minis with Mac OS X Lion 10 (64 bit), 16 GB RAM and 2.7 GHz Intel Core i764-bit.

### Reasoners Used

For all our experiments, we use four OWL reasoners that implement the OWL API interface: HermiT 1.3.8, Pellet 2.3.1, JFact 1.2.3 and FaCT++ 1.6.3. All four are among the most heavily used reasoners^13^ for OWL 2 DL. HermiT uses a hyper-tableau approach, while the other three reasoners employ standard Enhanced Traversal/tableau-based techniques. Despite the differences of hyper-tableau and normal tableau, both fit under the model outlined in Sect. 5.3. For the remainder of this paper, a subsumption test is a test either triggered by an Enhanced Traversal/tableau reasoner *or* a hyper-tableau reasoner. The reasoners have been modified for the benchmark: When a subsumption test is conducted, the start and end timestamps, the sub and super class under consideration, and the result of the test are recorded, see Sect. 5.3.

As of 2014, the new flagship reasoner of the DL community is Konclude [37], and every work around OWL reasoners must have a good reason for excluding it from its experiments. In our case, Konclude had to be excluded for three main reasons. Firstly, at the time of running the experiments, there was no really convenient way to interacting with Konclude through the OWL API. The only way was through OWLlink, which required the user to first start a Konclude server instance outside of the virtual machine the experiment was run in. This way of interacting with the reasoner created some (often parsing related) bottlenecks, and inconveniences in terms of experimental setup (killing the server after each classification, waiting for the operating system to free the port it was run on, and more). Secondly, implementing the Reasoner Stage Benchmark would have required effort from the Konclude developers. As we were only just developing the Stage Benchmark, it was only feasible to interact with developers more local to us (FaCT++). Lastly, and most importantly, our approach to time measurement was at the time of writing not robust for parallel implementations such as the ones found in Konclude. It is likely for example that the use of System.nanoTime() is problematic when benchmarking Konclude.

While we can use this approach to compare results for each reasoner, care has to be taken when interpreting the results of comparisons between reasoners because of implementation particularities. For example, some reasoners might apply graph-based methods to determine subsumptions up-front, and other might have them more tightly intertwined with their (hyper-) tableau engine. In order to choose where exactly to measure, we either asked the developers directly (JFact, FaCT++) or were guided by benchmarking code already present (progress monitors for debugging in HermiT and Pellet). Because we are interested in real life behaviour, we allowed the reasoner to fall into any internal state it normally would, such as the deterministic part of HermiT for Horn-SHIQ or Pellet’s internal EL-Reasoner. That said, we do not claim to time all subsumptions a reasoner determines because they are also determined by nested consequence-based approaches or during pre-processing using structural approaches. We are confident, however, that we capture all tests determined by actual calls to the tableau engine *during traversal*.

JFact is a Java-port of the C++-based FaCT++, and was chosen mainly to analyse the similarities and differences with FaCT++. While the algorithms are strictly the same, JFact usually is a couple of months behind FaCT++ in terms of up-to-dateness, and therefore not always equal in results. Because it is easier to integrate data types in Java, JFact does support more of them, and may therefore process certain ontologies that FaCT++ rejects.

### Experimental Pipeline

#### RQ1: Landscape of Subsumption Test Hardness

For the first experiment, we executed, for each reasoner, a single run across the entire corpus, with a timeout of 60 min per run.^14^ Note that we included every ontology in the corpus, including the ones not strictly in OWL 2 DL (53), but isolate them in the analysis. The reason for that is that these ontologies do form part of the landscape, and reasoners are used on them. The main sources of violations were uses of reserved vocabulary (37% of all violations across the corpus), illegal punning (32%) and uses of datatypes not on the OWL 2 datatype map (11%).^15^

#### RQ2: Effect of Modularity

For the second experiment, we selected a set of ontology-reasoner pairs for which, according to the results of the previous experiment, at least one subsumption test was measured that took longer than 100 ms. This bound is set for convenience: it results in a nice sample size (see Sect. 6.2.2), it is easy to memorise and it is clearly non-trivial. Because of the various claims we have with respect to modules, we also excluded ontologies that do not fall under OWL 2 DL. *Runtime limitations* forced us to exclude the NCIt from the sample, due to the extreme number of measured subsumption tests (JFact 751,907 tests, Pellet 461,831, FaCT++ 605,481). In order to answer Question 2, we classified tests by analysing how modularity affects their hardness. First we identified all super and sub-module combinations $\mathcal {M}_1,\mathcal {M}_2$ as follows: We obtained random cumulative subsets from the ontologies in our narrowed-down sample, similar to Gonçalvez et al. [12], with 16 slices. In a nutshell, the approach works as follows: (1) We randomly sample $\frac{1}{16}$ of the ontology (slice 1), then (2) randomly sample another $\frac{1}{16}$ and adding it to slice 1, making the second slice a random $\frac{2}{16}$ of the ontology. Then we add another random $\frac{1}{16}$ to the second slice, resulting in the third slice, and so on.

For each subset $\mathcal {S}\subseteq \mathcal {O}$ sampled, we obtained a corresponding module $\mathcal {M}_{\widetilde{S}}:=\bot -mod(\widetilde{\mathcal {S}},\mathcal {O})$. Module properties ensure, given two subsets $S_1,S_2$ with $S_1\subseteq S_2$, that $\mathcal {M}_{\widetilde{S_1}}\subseteq \mathcal {M}_{\widetilde{S_2}}$ [32]. The module of the 16th $\frac{1}{16}$, $\mathcal {M}_{\widetilde{\mathcal {O}}}$, corresponds to the whole ontology. We call this nested set of modules a *path*. Note that the modules are on average 40% larger than their respective subsets, which will give us a good sample of relatively large modules with hopefully hard subsumption tests. Each of the modules obtained was classified three times (i.e. in three independent runs) by each reasoner.^16^ Given a path $\mathcal {M}_1\subseteq \mathcal {M}_2\subseteq$ ... $\subseteq \mathcal {M}_n$, we call $\mathfrak {P}$ the set of all pairs $\mathcal {M}_i, \mathcal {M}_j$ with $i<j$. Given a pair $\langle \mathcal {M}_1,\mathcal {M}_2\rangle \in \mathfrak {P}$ and a reasoner $\mathcal {R}$ we compare $time(C(\mathcal {M}_1,\mathcal {R},i))$ with $time(C(\mathcal {M}_2,\mathcal {R},j))$ and compare subsumption test duration $time(ST(A,B,\mathcal {M}_1,\mathcal {R},i))$ with $time(ST(A,B,\mathcal {M}_2,\mathcal {R},j))$.

To make the following discussion easier, we introduce the following abbreviations:

$$\begin{aligned} \mathsf {min}(A,B,\mathcal {R},\mathcal {M}):= & {} \mathsf {min}\{ST(A,B,\mathcal {R},\mathcal {M},i) \mid i\in \{1,2,3\}\}\\ \mathsf {max}(A,B,\mathcal {R},\mathcal {M}):= & {} \mathsf {max}\{ST(A,B,\mathcal {R},\mathcal {M},i) \mid i\in \{1,2,3\}\} \end{aligned}$$

Every pair of measurements has a *tendency*, a *magnitude* and a *degree of stability*, see Table 1.

**Table 1:** Dimensions of subsumption test hardness *change under modularity*

Feature	Value	Measurement
Tendency	Easier	Mean hardness change $<-$5%
	Harder	Mean hardness change > 5%
	Neutral	Absolute mean hardness change < 5%
Magnitude	High	Absolute mean hardness change > 50%
	Medium	Absolute mean hardness change 5–50%
	Low	Absolute mean hardness change < 5%
Stability	Clear cut	All measurements same tendency
	High	Overlap of ranges of measurements < 10%
	Low	Overlap of ranges of measurements > 10%

We call the tendency *easier* if a test is easier in the super-module than in the sub-module (potentially pathological), *harder* the respective reverse, and *neutral* if the mean measurement difference does not differ by more than 5% between sub- and super-module. *High magnitudes* are changes above 50% (the test is more than 50% harder/easier in the super-module compared to the sub-module), medium magnitudes are changes between 5 and 50% and low changes are absolute changes below 5%.

An effect can be of three *degrees of stability*: *clear cut*, *high* or *low*. Given $\mathcal {O}$, two modules $\mathcal {M}_1\subset \mathcal {M}_2\subset \mathcal {O}$, and two subsumption tests $ST(A,B,\mathcal {M}_i,\mathcal {R},\cdot ))$, we consider three cases of change stability:

- *Clear cut*, if either
  - $$\mathsf {max}(A,B,\mathcal {R},\mathcal {M}_1) < \mathsf {min}(A,B,\mathcal {R},\mathcal {M}_2)$$ or
  - $$\mathsf {max}(A,B,\mathcal {R},\mathcal {M}_2)< \mathsf {min}(A,B,\mathcal {R},\mathcal {M}_1)$$
- *High*, if either
  - the interval $(\mathsf {min}(A, B, \mathcal {R}, \mathcal {M}_i),\mathsf {max}(A, B,\mathcal {R},\mathcal {M}_i))$ is contained in the interval $(\mathsf {min}(A, B, \mathcal {R}, \mathcal {M}_j),\mathsf {max}(A, B,\mathcal {R},\mathcal {M}_j))$ or
  - the overlap of the ranges of $ST(A,B,\mathcal {M}_1,\mathcal {R},i)$ and $ST(A,B,\mathcal {M}_2,\mathcal {R},j)$ is less than 10% of the range of all six measurements, i.e., if
    $$\begin{aligned} \begin{array}{ll} &{} \mathsf {min}(\{\mathsf {max}(A, B,\mathcal {R},\mathcal {M}_1 ),\mathsf {max}(A, B, \mathcal {R}, \mathcal {M}_2)\}) \\ &{}-\;\mathsf {max}(\{\mathsf {min}(A, B,\mathcal {R},\mathcal {M}_1 ),\mathsf {min}(A, B, \mathcal {R}, \mathcal {M}_2)\}) \\ < 0.1 \times &{}( \mathsf {max}(\{\mathsf {max}(A, B,\mathcal {R},\mathcal {M}_1 ),\mathsf {max}(A, B, \mathcal {R}, \mathcal {M}_2)\}) \\ &{} - \; \mathsf {min}(\{\mathsf {min}(A, B,\mathcal {R},\mathcal {M}_1 ),\mathsf {min}(A, B, \mathcal {R}, \mathcal {M}_2)\})) \end{array} \end{aligned}$$
- *Low*, if the stability is neither clear cut nor high.

**Fig. 1:**
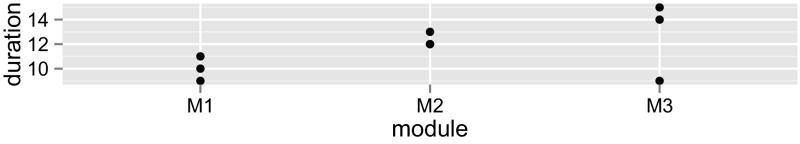
An example for multiple measurements taken for a single subsumption test across three modules $\mathcal {M}_1\subset \mathcal {M}_2\subset \mathcal {M}_3$

Note that cases of *neutral tendency* have high stability if both sets of measurements have a variation coefficient of less than 5%. The example in Fig. 1 shows a clear cut hardness change from module $\mathcal {M}_1$ to $\mathcal {M}_2$, but one with low stability from $\mathcal {M}_2$ to $\mathcal {M}_3$. We sometimes refer to changes of more than 5% as significant.

### The Phase Benchmark

Most OWL Reasoner benchmarks, especially those focused on classification, determine how long it takes the reasoner to execute the service for a given input. Few benchmarks distinguish between different stages outside the actual reasoning. We propose a model of monolithic reasoning that distinguishes the following five stages for the process of classification: (1) Preprocessing, (2) initial consistency check, (3) pre-traversal optimisations, (4) traversal, (5) postprocessing. The reasoning systems we analysed in our framework all follow that model, and we believe that most OWL DL classifiers do. The second core aspect of the framework is the *recording of subsumption tests*. We restrict ourselves to calls to the Tableau implementation of the reasoner, and ignore subsumptions determined by other means (e.g. nested consequence-based procedures). From an implementation perspective, the framework currently has to be hard wired into the code (a single static Java class). While we did this ourselves for the Java-based systems in our study, we collaborated with the developer of FaCT++ in order to extend the interface to merely flushing out textual information that we then later parsed back into our analysis framework. All measurements reflect wall clock time, to avoid the confusion provided by multi-threaded implementations. We also believe that wall clock time more realistically reflects what end-users are interested in, despite being a potential source of experimental error.

## Results

Percentages in this section are subject to uniform rounding. The measures used in this section (OCT, HST, SST) were described in Sect. 4.3. We group subsumption test hardness into the following bins: Very Hard (>100 s), Hard (>10 s, $\le$100 s), Medium Hard (>1 s, $\le$10 s), Medium (>100 ms, $\le$1 s), Medium Easy (>10, $\le$100 ms), Easy (>1, $\le$10 ms), Very Easy (>100 $\upmu$s, $\le$1 ms), Trivial ($\le$100 $\upmu$s).

Out of the 1320 attempted classification runs (4 reasoners and 330 ontologies), 1109 (84%) completed successfully. Of the 330 ontologies, 240 ontologies (81%) were dealt with by all four reasoners within the 60 min timeout, another 24 by 3 reasoners, 28 by 2, 21 by just 1 and 17 by none of the four reasoners, see Table 2. Since we have considered OWL Full ontologies in this particular survey, we present the numbers of successfully classified ontologies broken down by OWL profile category and whether they triggered subsumption tests.^17^ Table 2 also serves as a binning for further analysis later on, so it makes sense to study it carefully before moving on. Note that out of the 17 ontologies that no reasoner classified within the timeout, none fell under OWL 2 EL, QL or RL.

**Table 2:** Binning of all 330 ontologies by success category and test category

Test	All reasoners successful				Some reasoners successful				All Unsuccessful			
All	240				73				17			
Profile	All	DL	Prof	Full	All	DL	Prof	Full	All	DL	Prof	Full
All Prof.	240	75	146	19	73	37	8	28	17	11	0	6
No test	152	13	136	3	25	4	8	13	-	-	-	-
Some test	41	23	10	8	48	33	0	15	-	-	-	-
All test	47	39	0	8	-	-	-	-	-	-	-	-

*Prof* stands for profiled (EL, QL, RL), *DL* stands for pure DL (OWL DL, excluding profiled), and *Full* stands for OWL Full (excluding OWL DL)

FaCT++ completed 268 ontologies in total (81%), HermiT 284 (86%), JFact 270 (82%) and Pellet 287 (87%). Reasons for failure include hitting the timeout, unsupported datatypes, and ontology inconsistencies. Table 3 (“Appendix”) shows a detailed account of that.

**Table 3:** Detailed account of successes and failures, as they were reported in the form of Java Exceptions

	FaCT++	HermiT	JFact	Pellet
Illegalargument	1	16	1	1
Unsupportedoperation	0	0	0	1
Owlreasonerruntime	10	0	0	0
Reasonerinternal	23	0	29	0
Nullpointer	0	0	5	0
Arrayindexoutofbounds	0	0	0	1
Concurrentmodification	0	0	0	1
Inconsistentontology	3	2	0	7
Malformedliteral	0	1	0	0
Unsupporteddatatype	0	14	0	0
Numberformat	0	0	1	0
Timeout	4	3	2	24
Unknown	21	10	22	8
Success	268	284	270	287

Unknown items are most likely those that had to be terminated by the test framework, thereby not leaving an explanation of failure

In order to improve our understanding of how different reasoners compare, we present in Table 4 the number of ontologies broken down by (1) which reasoner dealt with them and (2) which reasoners they caused to trigger a subsumption test. As (1) has a strong impact on (2), we decided to present for (2) only the break-down of the 240 ontologies that all four reasoners successfully classified. (2) will be discussed in more detail later on. Note that with respect to (1) there is almost no discernible pattern of reasoner factions (i.e. groups of reasoners that behaved in a similar fashion). A large proportion of all possible combination is represented, including unlikely dominant combinations such as JFact, FaCT++ and HermiT, given that the strongest faction, Pellet all by itself (and the fact that it has the most completed classifications of all four reasoners), appears to be a sign that Pellet is quite resistant to failure.

**Table 4:** Left: Combinations of reasoners to successfully classify an ontology out of all 330 ontologies. Right: Combinations of reasoners to fire tests out of the 240 ontologies that all reasoners dealt with

J	F	P	H	freq	J	F	P	H	freq
X	X	X	X	240					152
				17	X	X	X	X	47
		X		13				X	18
X	X		X	9	X	X			10
X		X	X	8	X	X	X		7
X		X		7	X	X		X	5
		X	X	7		X			1
	X		X	6					
			X	6					
	X	X	X	5					
	X	X		5					
X			X	3					
X	X	X		2					
X				1					
	X			1					

### Role of Subsumption Testing in Classification

In the following, we describe our observations that help us in addressing RQ1. We break down the main question into the following sub-questions before we summarise the most important observations in our discussion.

1. **Which ontologies cause reasoners to trigger subsumption tests?** First, we will narrow down how many ontologies are affected, and what kinds of ontologies are affected. Then we will take a brief look at the differences between reasoners.
2. **What are real subsumption tests like?** We will see how many tests are generally fired and we will analyse them in terms of hardness and the differences between positive and negative subsumption tests, before we look in more depth at the differences between the four reasoners.
3. **What is the Contribution of Subsumption Test Hardness To Classification Performance?** We will take a look at the SST/OCT ratio (see Sect. 4.3), determine the effect of very hard tests and the potential gain involved making these tests easier or less numerous.
4. **What are the shared characteristics of ontologies with a high impact factor?** For those ontologies for which we determined that subsumption testing plays a big role, what are they like? Are there any obvious structural characteristics that these ontologies share?
5. **What is the performance of traversal algorithms?** In this ancillary question, we will look at how traversal algorithms fare against the $n*log(n)$ and naive $n^2$ upper bounds.

#### Which Ontologies Cause Reasoners to Trigger Subsumption Tests?

Our first observation is that 152 (**46.1%) of the ontologies were classified by all four reasoners and did not trigger a single subsumption test**,^18^ see Table 2. Another way to put it (from the perspective of successful classifications): only 33% of all ontology-reasoner pairs in the set of successful classifications involved one or more calls to the tableau engine.^19^ *All four* reasoners fired tests in 47 of the ontologies. 136 ontologies caused at least one reasoner to conduct a subsumption test.^20^ Out of these 136 ontologies, only 10 ontologies fall under one of the profiles, all of which are pure OWL 2 EL (i.e., they neither fall under OWL 2 QL nor OWL 2 RL). The remaining 144 ontologies falling under one of the profiles did not trigger any tests at all.^21^ 70% of the above 136 ontologies are pure DL (see Sect. 4.3), that means of considerable expressivity.

*Differences across reasoners*. Overall, FaCT++ did not trigger any subsumption tests in 177 cases (66% of successful classifications), HermiT in 180 (63%), JFact in 182 (67%), and Pellet did not fire a subsumption test during 209 (73%) successful classifications.

To get a picture about the agreement between the reasoners on whether calls to the tableau engine are required at all, we will zoom in on the 240 ontologies that all four reasoners successfully dealt with. Table 4 (right side) shows how reasoners differ in opinion whether tests are necessary. The two largest factions in cases of disagreement are HermiT all by itself (18 ontologies) and JFact /FaCT++ (10 ontologies). The second faction is perhaps explained by the architectural similarity between FaCT++ and JFact, see Sect. 5.1. 63% of the 240 ontologies did not trigger a test by any of the reasoners. In 8% of the ontologies, only one reasoner (mainly HermiT, see above) triggered a test, in 4% two reasoners, in 5% three reasoners and in 20% all four reasoners. Note that all cases for which there is no agreement on whether tests are necessary or not essentially indicate missed opportunities for optimisation (at least one reasoner managed to classify without firing a test).

Table 5 shows how these 240 ontologies are distributed across the size bins (for an explanation of the binning, see end of Sect. 4.2). The main observation to take from that is that most disagreements (proportionally) happen among the medium and large ontologies (between 100 and 10,000 axioms). We define *agreement* as the cases where either all reasoners or no reasoners fired a test, and *disagreement* the respective reverse.

**Table 5:** Contingency table showing ontology size to number of reasoners ($|\mathcal {R}|$) to fire one or more subsumption tests

$|\mathcal {R}|$	Empty	Very small	Small	Medium	Large	Very large	Huge
1	0	0	2	11	5	1	0
2	0	0	0	3	7	0	0
3	0	0	1	8	2	1	0
4	0	0	1	24	15	6	1
0	0	2	21	58	52	17	2

The top three rows reflect disagreement (see text), the bottom two agreement between the reasoners

Table 6 shows how the distribution of ontologies with respect to their expressive power (or a proxy thereof) and the number of reasoners firing tests while dealing with them. Only a handful of ontologies that fall under the OWL 2 RL, EL or QL profiles (10) force some reasoner to trigger a test. All 10 cases are caused by JFact and FaCT++ (always both). For 18 of the 19 cases that all reasoners successfully dealt with and only one of the four reasoners firing a test, that reasoner was HermiT; only in one case, FaCT++ fired a test, while the other reasoners did not.

**Table 6:** Contingency table showing ontology OWL profile bin to number of reasoners ($|\mathcal {R}|$) to *fire a subsumption test*

$|\mathcal {R}|$	OWL Full	Profiled	Pure DL	All
0	3	136	13	152
1	7	0	12	19
2	0	10	0	10
3	1	0	11	12
4	8	0	39	47

#### What are Real Subsumption Tests Like?

Across all 1,109 successful classifications we measured 2,671,896 subsumption tests, 54,934 out of which turned out positive (2.06%) and 2,616,962 out of which were negative (97.94%), see Table 7. Positive tests account for only between 0.12% (Pellet) and 2.61% (FaCT++) of the overall number of tests (HermiT 2.12%, JFact 2.49%). This low ratio is not surprising, as the number of negative subsumptions vastly outweigh the number of positive ones in any typical ontology, but we do not currently know why the ratio for Pellet is much lower than the one of the others, despite their architectural similarities. Subsumption test hardness varies widely: while most subsumption tests are easy (e.g., half of all tests take less than 481 $\upmu$s for HermiT and less than $71\,\upmu$s for FaCT++), the hardest ones take over 3 min.

**Table 7:** Subsumption test hardness: Descriptive Statistics (unit $\upmu$s), number of positive ($|\mathcal {ST}|+$) and negative ($|\mathcal {ST}|-$) tests, by reasoner $\mathcal {R}$

$\mathcal {R}$	Min.	Q1	Med.	Avg.	Q3	Max.	$|\mathcal {ST}|+$	$|\mathcal {ST}|-$
F	2	46	71	7,519	111	2,352,000	24,286	905,011
H	48	418	481	17,390	570	198,900,000	1,911	88,387
J	1	48	88	1,127	169	45,920,000	28,103	1,100,972
P	23	175	246	825	365	35,060,000	634	522,592

**Fig. 2:**
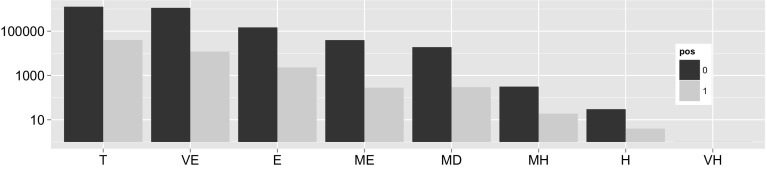
Counts of subsumption tests for each hardness bin (log scale), distinguished by positive (1) and negative (0) tests

Figure 2 shows the distribution of subsumption tests across the hardness bins. Keeping in mind that the figure is presented with a logarithmic scale, we can see that by far the majority of tests are negative/trivial or negative/very easy, i.e. 90.18% of all measured test take less than a millisecond.

**Fig. 3:**
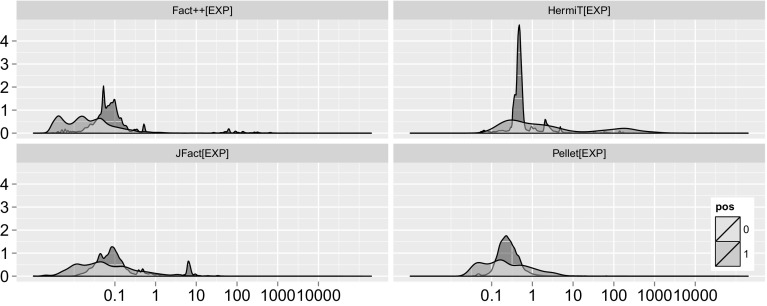
Kernel density plot of subsumption tests for each hardness bin (x:log scale, milliseconds), distinguished by positive (1) and negative (0) tests. Subsumption tests across entire experiment

To get a better picture of the distribution of tests, see Fig. 3. A striking feature of HermiT appears to be that negative tests are densely centred around approximately half a millisecond, while all the other reasoners appear to have a larger spread of negative test hardness. As a reminder, positive subsumption tests correspond to negative satisfiability tests, which have often been assumed to be harder because in the worst case, all branches need to be explored to verify the unsatisfiability. Another observation to note is that FaCT++ and JFact find a number of negative tests hard (and no positive ones), while HermiT triggers some tests that are hard and turn out positive, and few hard negative ones.

**Fig. 4:**
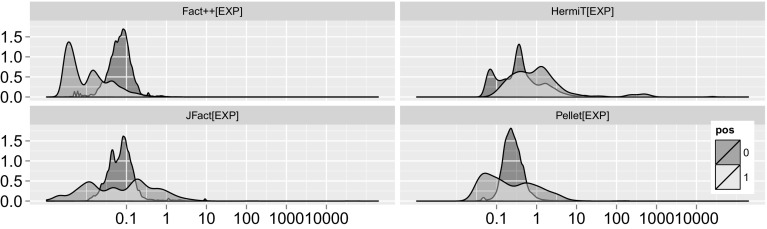
Kernel density plot of subsumption tests for each hardness bin (x:log scale, milliseconds), distinguished by positive (1) and negative (0) tests. Subsumption tests across 39 OWL 2 DL ontologies with tests triggered by all four reasoners

In order to better understand how reasoners differ in terms of subsumption test hardness, we will look at the *39 pure DL ontologies* processed by all four reasoners in more detail. The distribution of subsumption test hardness for those ontologies is shown in Fig. 4. Only HermiT fires a handful of positive tests harder than 100 ms. In terms of negative tests, only Pellet and HermiT have tests harder than 1 s, and only a handful. Another observation to take away is that towards the very easy part of the figure (less than 0.01 ms), we find for FaCT++, JFact and Pellet more positive than negative tests. For these three, negative tests also seem to be approximately (log-)normally distributed. Whether the differences for HermiT are due to the architectural differences is up for further investigation.

To answer the question whether positive tests or negative tests are generally harder, the choice of the measure of central tendency is crucial. As we can see in Fig. 4 the distribution of test hardness is in most cases not even log normal, in some cases they appear even multi-modal (see positive FaCT++ tests). The high skew renders the mean a quite deceptive tool to compare the hardness of positive and negative tests. In terms of median, which is quite insensitive to outliers but more applicable for these kinds of distributions, we cannot learn much. Because we need to explore all branches of the tableau, positive tests are typically considered harder than negative ones. Surprisingly, for FaCT++, tests resulting in non-subsumptions are generally harder than positive tests (10 times on average, using median), for HermiT 2.6 times and for Pellet 1.5 times. Only JFact, finds positive tests 1.05 times harder than negative tests. As these aggregations are largely dominated by the fast tests, it makes sense to take a closer look at the harder ones. Since hardest test measurements are quite sensitive to experimental error, we focus our attention on the 90th quantile. Here, the picture appears almost reversed. For FaCT++, negative tests are still 2.3 times harder than positive ones. For HermiT *positive* tests appear 2.03 times *harder* than negative ones, for Pellet 3.2 times and for JFact 5.5 times, which corresponds much more to what we would expect. One interesting observation is the striking similarity between Pellet and JFact. Both appear to have a wide range of positive tests, and a large “pillar” of negative tests almost at the center of it, where there is also a small downward bulge from the positive tests.^22^ It is part of future work to explain why some reasoners find negative, and some reasoners positive tests harder; any attempts at an explanation given the current data would remain speculation.

**Fig. 5:**
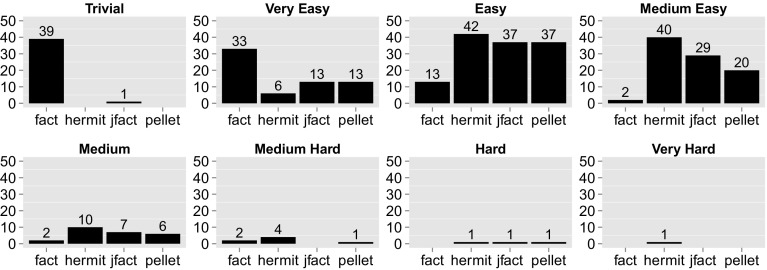
Ontologies in each hardness category. The *x-axis* represents the number of ontology in each bin. Bin classification according to hardest subsumption test

To determine how prevalent individual hard reasoning problems are, it makes sense to group the ontologies in our corpus by the *hardest test* fired. Figure 5 shows the entire corpus binned by hardest test. The most important observation to make here is the rarity of ontologies with tests that take longer than a second (medium hard bin and above). The dominating cases are ontologies whose hardest tests range between 1 and 100 ms. Note that this observation of low hardness contradicts observations made by Gonçalvez et al. [12]; many of the tests they measured were harder than 100 ms. However, satisfiability checks (in their experiments) were performed in a blackbox fashion from outside the process of classification, which may have introduced some overhead due to the particular implementation of the satisfiability method of a given reasoner.

#### What is the Contribution of Subsumption Test Hardness To Classification Performance?

**Fig. 6:**
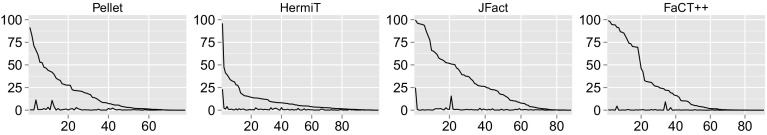
Impact of SST on classification time by reasoner in %. *Low line* hardest individual test; *high line* sum of all tests; *x-axis* ontologies; *y-axis* contribution in %. Note that x-axis values mean something different for each reasoner: The set of ontologies for which a given reasoner triggered subsumption test, ordered by ratio of SST to OCT

In Fig. 6 we show the contribution of all subsumption tests and the contribution of the hardest test to the overall classification time (OCT), broken down by ontology and reasoner.

While the sum of all subsumption tests dominates the OCT only in a few cases (very few for HermiT, more for JFact and FaCT++), it occasionally accounts for more than 80%. Only 1 ontology has more than a 50% contribution of total SST on OCT for Hermit, 7 for Pellet, 19 for FaCT++ and 23 for JFact. Very rarely can we observe a single test accounting for more than 10% of the OCT. The maximum impact for a single test by FaCT++ is 9.2%, Pellet 11.3%, HermiT 23.1% and JFact 24.8%. According to our notion of *strong impact* (see Sect. 4.3) we count 3 for ontologies HermiT, 12 for Pellet, 21 for FaCT++ and 26 for JFact.

Trivial and very easy tests dominate by far in terms of number, but they are *not responsible for the majority of the impact* on OCT. Table 8 breaks down the overall impact of tests belonging to a particular hardness category across the entire corpus (first row), compared to the subset of 39 DL ontologies that all four reasoners processed (second row). For example, the sum of all negative (0) hard (H) tests across all 39 DL ontologies is 10.9 min. Negative medium (M) and negative medium-easy (ME) tests, i.e. tests between 10 ms and 1 s, have by far the greatest impact: Out of the 171.3 min reasoners spend doing subsumption tests across the whole experiment, 90 min (53%) was spend on negative (0) tests of medium (M) difficulty (a further 31.6 min (19%) was spend on tests of medium-easy (ME) difficulty). Interestingly, among the 39 pure OWL 2 DL ontologies that all four reasoners dealt with (second row), the dominating type of tests are hard negative tests (tests between 10 and 100 s).

**Table 8:** Aggregated sum of all tests (in min) across the whole experiment by hardness category

T		VE		E		M		ME		MH		H		VH
0	1	0	1	0	1	0	1	0	1	0	1	0	1	0
1.2	0.0	5.1	0.1	12.2	0.1	90.0	1.4	31.6	0.2	10.4	0.5	12.1	3.1	3.3
1.1	0.0	3.3	0.0	1.2	0.0	1.6	0.2	0.4	0.0	0.4	0.0	10.9	3.1	3.3

The first row accounts for *all* subsumption tests measured as part of this experiment, the second corresponds to the 39 pure DL ontologies for which all four reasoners triggered subsumption tests. The abbreviations (T, VE, .., VH) correspond to the hardness bins from Trivial to Very Hard (see Sect. 6.1.3). 0 or 1 indicates whether the test turned out to be positive (1) or negative (0). For example, the sum of all negative (0) medium (M) subsumption test times measured as part of this experiment was 90 min; when considering only the aforementioned 39 ontologies, the sum was (only) 1.6 min

To understand what this means, consider this case: an optimisation that reduced the mean hardness of subsumption tests by 50% would, taking into account only those ontologies for which tests were triggered, reduce the overall classification time on average by 14.2%. There are however differences between the reasoners: for JFact, the overall classification time could be reduced by 21.3%, for FaCT++ by 19.4%, for Pellet by 11.4% and for HermiT only by 5.8%. If we only take into account the 39 ontologies for which all four reasoners triggered tests, we would have a similar picture: the OCT would be reduced on average by 16.6% for JFact, by 14.02% for FaCT++, by 12.03% for Pellet and by 6.9% for HermiT.

#### What is the Profile of Ontologies with a High Impact Factor?

Only 10 unique ontologies across the corpus trigger *hard tests* (harder than 1 s) by any one reasoner, 8 of which are pure DL (all beyond $\mathcal {ALC}$), and 2 of which are in OWL Full. Out of the 8 OWL 2 DL ontologies, 4 are only of medium size (between 100 and 1000 axioms), 3 are large (between 1000 and 10,000 axioms) and 1 ontology has more than 100,000 axioms. 7 of the 8 ontologies involve inverse roles, and all 8 ontologies involve role hierarchies (either languages $\mathcal {H}$ or $\mathcal {R}$).

Among the 38 ontologies for which at least one reasoner registered a *strong impact* of subsumption testing on the performance of the overall classification time, 30 were pure OWL 2 DL, 2 fell under OWL 2 EL and 6 were OWL Full. The ontologies are scattered across most size ranges: 4 have more than 10,000 axioms, another 20 more than 1000 and 14 ontologies have less than 1000 axioms. 33 of these 38 ontologies contain inverse roles, and 35 contain role hierarchies, 19 out of which contain more complex role-related modelling ($\mathcal {R}$). More than half contain nominals.

#### What is the Performance of Traversal Algorithms?

**Fig. 7:**
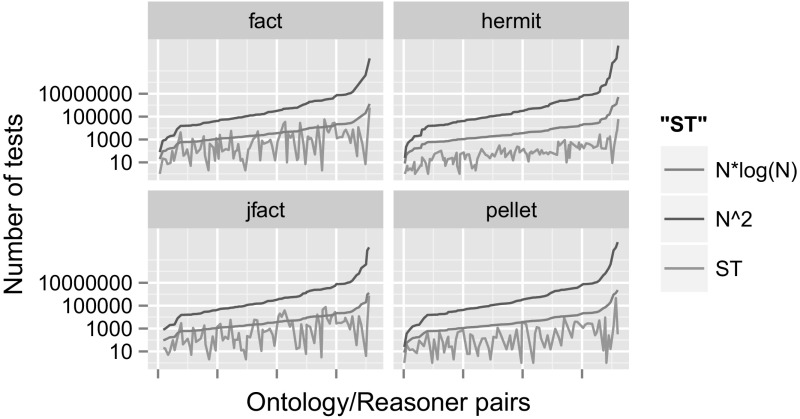
Subsumption tests carried out in relation to a naive $N^2$ upper bound and an $N \log (N)$ upper bound, ordered by *N*, the number of names in $\widetilde{\mathcal {O}}$ (y:log scale)

Current traversal algorithms appear to be mostly effective, see Fig. 7, in particular the ones implemented in Pellet and HermiT: they exceed the $N*\log (N)$ upper bound only once, and no reasoner comes even close to the naive $N^2$ upper bound (*N* being the number of class names in $\widetilde{\mathcal {O}}$). We discussed both of these upper bounds in Sect. 2. Exceeding the $N*\log (N)$ upper bound suggests that the reasoner was faced with a poly-hierarchy, i.e. an ontology involving concepts with multiple super-classes. Exploring the exact relationship between the number of triggered subsumption tests and the shape of the class hierarchy is part of future work.

#### Discussion

A first answer to Question 1 is that (tableau) subsumption testing does not contribute at all to classification time for a substantial number of ontologies. We have established a *empirical lower bound* for BioPortal ontologies that do not involve subsumption testing at 46%. This lower bound is even slightly naive because (1) there are most likely a number of ontologies that do not involve tests among the unsuccessfully classified ones and (2) only 33% of all ontology-reasoner pairs involved tests.

The currently secured *lower bound* for ontologies actually requiring subsumption testing lies at 14% (i.e. the 47 out of 330 ontologies for which all four reasoners triggered a test). Note that, while this might seem like a very low number, these might be the 50 or so ontologies in the world that are hard and *matter*, and thus worth optimising for. As a side note, the low numbers of tests for HermiT and Pellet can perhaps be explained by their internal alternative deterministic engines (for example internal $\mathcal {EL}$-reasoners), see Sect. 5.1.

It is quite interesting that only 10 out of those 146 ontologies that all reasoners processed caused at least one reasoner to fire a test—all of which are *pure* OWL 2 EL. Ontologies of the OWL 2 RL or OWL 2 QL family, or less expressive ontologies, did not cause any reasoner to actually fire a test. This suggests that for OWL 2 RL and OWL 2 QL ontologies at the very least, the application of modular techniques must be strictly motivated by a different argument than test avoidance or test easyfication. Another potentially interesting observation is that ontologies involving hard tests generally seem to contain rich role-level modelling, most prominently inverses and role hierarchies.

*Subsumption test hardness rarely has a strong impact* on classification performance. According to our threshold of “strong impact” at 40% of the overall classification time, FaCT++ encountered impactful ontologies 7.8% of the time, JFact 9.6% of the time, Pellet 4.2% of the time and HermiT only in 3 out of its 284 successful classifications (just around 1.1%). This, and taking into account the low absolute potential performance gains as described above, creates a *case against modular reasoning techniques* motivating them the way we currently do (avoidance, hardness reduction). These results do not affect modular techniques motivated differently (like partial reclassification in incremental reasoning or expressivity reduction for partial classification by cheaper algorithms such as MORe  [31]).

The most important *threat to the validity* of the results presented in the previous section is the tight (albeit necessary) *timeout of 60 min*, a limitation that pervades most experiments presented as part of this work. It might well be that the 211 missing ontology-reasoner pairs all triggered subsumption tests, even hard ones, before they timed out, or would have triggered tests if they were not rejected for an unsupported datatype. In principle, these failed cases could create a very good case for modular reasoning. A second threat to validity is the *unavailability* of fine grained correctness benchmarks. In an independent study [22] we revealed at least 9 ontologies in our corpus for which there was disagreement between the reasoners. Incomplete reasoning could potentially skew the measurements (test counts and duration) significantly. OWL 2 Full ontologies are not dealt with uniformly by all reasoners; essentially anything can happen when reasoning with them (for example dropping axioms). This is why the survey does not lend itself to comparisons of the performance of the reasoners directly (beyond what has been presented).

### Sensitivity to Modularly Irrelevant Axioms

The previous sections were dedicated to studying monolithic traversal/(hyper-) tableau style classification across BioPortal, specifically for improving the understanding of the role of subsumption testing for OWL classification. In the following section, we will discuss RQ2. We will first describe general difficulties of measuring the “effect of modules” on subsumption test hardness, before we address the question and present our insights on *sensitivity to modularly irrelevant axioms*, or, in other words, the effect of modularity on subsumption test hardness, with respect to all four reasoners.

From the previous experiment, according to the process detailed in Sect. 5.2, 3 ontologies were selected for FaCT++, 4 for Pellet, 5 for JFact and 13 for HermiT for the following analysis (25 ontology-reasoner pairs). These ontologies have overall classification times ranging from 7 to more than 1200 s (median: 103.20, mean: 210.70). 16 modules were generated per ontology and classified, which led to *1,200* attempted classifications (3 runs per module, 25 ontology reasoner pairs) with a timeout of 60 min. Of these, 1,093 (91%) terminated successfully. By reasoner, that is 77.1% for FaCT++, 91.8% for HermiT, 98.4% for Pellet and 100% for JFact. Out of the 400 module classifications (16 modules, 25 ontology-reasoner pairs), we have 358 (89.5%) for which we have three successful classification runs. We exclude the remaining ones from the analysis.

#### Measuring the Effect of Modularity

There are two important factors that potentially threaten the internal validity of our results: (1) Experimental error (measurement variability caused by factors outside the program) and (2) stochasticity in the classification process. Both problems relate to different phenomena, but are usually not distinguishable from our experiment data alone.

Multiple runs of the same program do not usually lead to the same execution times. Experimental error (1) is a major cause for variance across timings. For example, system-level processes may kick in and add to the CPU load or varying times for memory allocation and garbage collection. Our measure for variability is (again) the *coefficient of variation* (COV). For each module-reasoner pair (MRP), we look at the variability of two distinct variables: overall classification time (OCT) and sum of all subsumption test times (SST).

The OCT of only 3 out of 358 (0.84%) module-reasoner pairs varies by more than 30%, of 12 (3.4%) MRP’s (including the ones above 30%) by more than 20%, and of 19 (5.3%) MRP’s by more than 10%. The module with the worst variation corresponds to a module taken from a $\frac{2}{16}$th of the Biotop ontology (727 logical axioms), classified by JFact (min = 38.49 s, max = 194.22 s). By reasoner, FaCT++ varies for OCT on average (median in brackets) by 0.79% (0.67%), JFact by 2.53% (0.88%), HermiT by 4.15% (2.33%) and Pellet by 4.32% (3.12%).

For SST, the reasoners vary as follows: FaCT++ 0.77% (0.66%), HermiT 9.02% (5.65%), JFact 2.61% (0.86%) and Pellet 5.43% (4.28%). A more detailed picture of the overall variation is given in Fig. 12 for both OCT and SST (“Appendix”).

Next we consider the variance of individual subsumption test hardness: across all 358 module-reasoner pairs, we measured the hardness of 2,438,983 distinct subsumption tests. The coefficient of variation is generally log10-normally distributed, but varies considerably across reasoners. On average, measurements deviate by as much as 13.22 and 13.96% for Pellet and HermiT, respectively, while measurements for JFact deviate by 3.5%, and for FaCT++ only 2.83% (see Fig. 13, “Appendix”). A more detailed breakdown can be found in Table 9.

**Table 9:** Variance of test measurements across reasoners (COV)

Reasoner	Mean	Min	25%	Median	75%	Max
Fact++	2.83	0	0.52	0.96	2.93	167.87
HermiT	13.96	0	6.70	13.13	19.85	172.50
JFact	3.54	0	0.73	1.35	2.94	169.07
Pellet	13.24	0	1.60	5.96	18.27	172.65

Not all variance is a consequence of experimental error. A varying subsumption test count is evidence for stochastic choices in the classification process. For example, a reasoner might require 10 tests to classify $\mathcal {O}$ in the first run, and 12 in the second. With 242 out of 358 cases (67.6%) showing differences in the number of subsumption tests measured across runs, variation is high. 20 module-reasoner pairs (5.6%) vary by more than 10% in the number of subsumption tests. These results cannot be generalised to the entirety of BioPortal (given the nature of the sample), but they suggest that at least some of the measurement variance is not a consequence of measurement error, but of stochastic effects. Another indication of stochastic effects in the classification process is the number of times a particular test was triggered across a number of runs, for example twice across three runs. Any number other than the exact number of runs would be evidence for some stochastic effect. In practice, we found only 105 (less than 0.005%) such tests that where *not* captured by the overall test count metric, i.e. that occurred in cases with varying overall test count.

Stochastic effects may not only be triggered by non-determinism in the tableau algorithm. FaCT++ for example does not reveal any evidence for stochastic effects during classification, while JFact does. This suggests that the source for stochastic effects might lie with data structures or methods in Java [7] (as FaCT++ is implemented mainly in C++). Unfortunately, the source of the stochasticity cannot be pinpointed by looking at test order alone: randomness in the implementation of the classification algorithm can induce changes in the exploration of non-deterministic branches of the tableau and the other way around. Effects of modularity may not be determined accurately in the presence of any strongly stochastic effects. In the following analysis, we will therefore often distinguish between those classifications for which we witnessed stochastic effects and those for which we did not.

Note that fluctuations in the number of tests or fluctuating (dis-)appearances of tests^23^ across runs establishes merely a lower bound for the number of cases that are potentially subject to stochastic effects. There are other signs, such as variation in the test order, that could be signs of stochasticity. Unfortunately, neither test count, (dis-)appearance nor order, are by themselves explanatory of classification hardness. They can neither, as pointed out before, point to the source of the stochasticity, nor can they be used to quantify the degree of stochasticity, i.e. the ratio of non-deterministic and deterministic choices. Due to our three run policy, there is a risk that we are falsely attributing changes in individual test hardness to modularity (e.g. reduced test hardness) that was actually caused by stochasticity. For example, consider a case with $\mathcal {M}_1\subset \mathcal {M}_2$ and six subsumption tests $ST(A,B,\mathcal {M}_j,\mathcal {R},i)$ that are measured three times (across three runs) in $\mathcal {M}_1$, and three times in $\mathcal {M}_2$, with all three measurements in $\mathcal {M}_2$ being easier than all three measurements in $\mathcal {M}_1$ (i.e. a pathological clear cut case). It could be the case that the difference in signature (1) induced a test order that triggered randomly other tests *before* $ST(A,B,\mathcal {O},\mathcal {R},i)$ that do most of the work (cached pseudo-models or similar) or (2) randomly led the tableau-reasoner into harder non-deterministic branches. In this case it can be argued that the easyfication, if it is merely due to test order, is *not* due to modularity, and beneficial non-determinism can be forced merely by changing the test order. For the remaining paper, in order to distinguish both cases in the analysis, we call a test measurement *potentially subject to stochastic effects* (1) if the test was either triggered during a classification with varying test counts across runs or (2) if the test appears/disappears across runs of the same classification (but the overall number of tests remains the same).

#### What Effect does Modularity have on Subsumption Test Hardness?

In order to investigate the effect of modularity on subsumption test hardness we sampled 30 sub-module super-module pairs $\mathfrak {P}$ from the 120 possible combinations as described in Sect. 5.2. Discarding unsuccessful classifications, we obtained data from 703 out of 750 possible comparisons. For result stability, we excluded all pairs that (1) were incomplete, i.e. we measured for either the sub or the super-module less than 3 runs and (2) the sub and the super-module were equal. This is because a test can be triggered for $C(\mathcal {M}_{sub},\mathcal {R},i)$ and not for $C(\mathcal {M}_{super},\mathcal {R},i)$, or for $C(\mathcal {M}_{super},\mathcal {R},i)$ and not for $C(\mathcal {M}_{sub},\mathcal {R},i)$. This fluctuating appearance is an indication that a test is subject to a stochastic effect. It is unclear how do deal with tests that are occasionally absent. For example, they can either be ignored, or they can be counted as zero duration. Our interest lies solely in determining whether individual tests triggered during classification are harder or easier in the sub-module. Therefore, we restrict our treatment of potential stochastic effects to isolating ontologies with evidence for stochastic effects from those without, as described in the previous section. The following analysis is conducted on the remaining 659 cases (87.87% of 750).

**Fig. 8:**
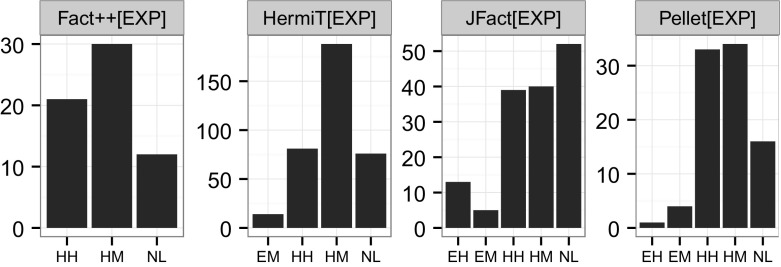
Hardness changes by reasoner: OCT. Bin labels x-axis: 1st letter: tendency (easier, neutral, harder), 2nd: magnitude (low, medium, high). Y-axis: number of comparisons. [EXP] indicates that reasoners have been modified to record subsumption tests for the purpose of this experiment

*Modules:* There are 39 cases (around 5.7%) where the OCT of the sub-module is higher than that of the super-module, and 173 (25.1%) where there is no significant change in hardness (less than 5% change), compare also Fig. 8.

From our 659 sub/super-module pair classifications, we obtained a total of 8,664,108 test comparisons. A comparison consists of

- two modules $$\mathcal {M}_{sub}\subset \mathcal {M}_{super}\subseteq \mathcal {O}$$,
- a test $$ST(A,B,\mathcal {M}_{x},\mathcal {R},\cdot ) $$,
- a set of measurements $\{ST(A,B,\mathcal {M}_{sub},\mathcal {R},i) \mid 1\subseteq i \subseteq 3\}$ and
- a set of measurements $\{ST(A,B,\mathcal {M}_{super},\mathcal {R},j) \mid 1\subseteq j \subseteq 3\}$.

Sometimes, we refer to such comparisons between sub- and super-module as “cases”.

**Fig. 9:**
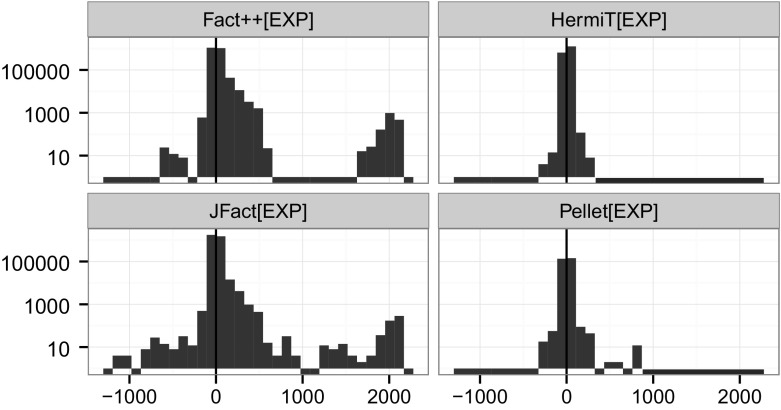
Histogram of change in hardness between sub and super-module. Counted are only such cases where the test was triggered in all three runs, both for the sub- and super-module

Overall, subsumption tests are easier in the sub-module than in the super-module across all reasoners and module pairs in the set. Tests triggered in the super-module are *harder by a factor of 2* (mean). The difference between reasoners however is large: While for FaCT++ tests in the super-module are harder by a factor of 6.18, test hardness for JFact (0.42), Pellet (0.257) and HermiT (0.075) are much less affected. If we further separate out ontologies with varying test counts as potential subjects to random effects, we find that HermiT finds tests *easier* in the super-module (−0.04 change), but harder when classifying ontologies with evidence for stochastic effects (0.08). Note that these changes are extremely low and cast doubt on the conjecture that HermiT could benefit from modularity in terms of subsumption test hardness reduction. It is quite possible that this low effect can be explained by architectural differences: the hyper-tableau implementation of HermiT mitigates the effect of algorithmic non-determinism for Horn ontologies by dealing with GCIs directly (rather than having to rewrite them into potentially costly, non-deterministic disjunctions) [9]. For Pellet we measured changes of 0.87 and 0.20 (potential stochastic effect) and for JFact 2.91 and −1.43 (potential stochastic effect). Table 10 presents an analysis of percentiles by reasoner. Note the potentially extreme effects of measurement error as a consequence of dealing with measurements in the microsecond area. The median, for all four reasoners, is around 0. Figure 9 confirms (note the log-scale on the y-axis) that the majority of tests are centred around 0, i.e. they do not change in hardness at all. Both JFact and FaCT++ however exhibit a bi-modal distribution, with at least one very distinct distribution of tests towards the hard end, i.e., tests that are significantly harder in the super- than in the sub-module. Interestingly, for almost all of these cases, the test in the sub-module differs from the test in the super-module by between 220 and 240 ms. This is quite a lot, given that the average difference between tests is around 0.56 ms. Most likely, an expensive subsumption test in the super-module was replaced by a cheap look-up in the sub-module.

**Table 10:** Summary for the change of subsumption test hardness from sub- to super-module

$\mathcal {R}$	Min	1%	5%	10%	25%	50%	75%	90%	95%	99%	Max
In cases with no evidence for stochastic effects in classification process											
F	$-$556.60	$-$24.55	$-$1.17	$-$0.14	$-$0.02	0.00	0.02	0.15	2.28	200.73	2155.73
H	$-$3.17	$-$2.18	$-$0.53	$-$0.40	$-$0.19	0.00	0.12	0.36	0.54	1.00	2.33
J	$-$1099.59	$-$24.24	$-$0.81	$-$0.20	$-$0.03	0.00	0.03	0.11	0.28	150.54	2149.22
P	$-$15.90	$-$0.67	$-$0.17	$-$0.11	$-$0.02	0.05	0.62	1.66	3.67	14.79	136.26
In cases with evidence for stochastic effects in classification process											
H	$-$264.02	$-$0.31	$-$0.18	$-$0.12	$-$0.03	0.05	0.16	0.28	0.36	0.53	278.37
J	$-$1083.87	$-$25.21	$-$19.18	$-$1.65	$-$0.08	$-$0.00	0.02	0.17	0.34	2.62	902.85
P	$-$287.54	$-$2.48	$-$0.72	$-$0.38	$-$0.09	$-$0.00	0.11	0.41	0.82	6.05	817.77

Measure: redefined normalised fold change. 0 means no change, 0.5 means a change of +50%

Given the low stability of many test measurements, we will present our analysis using our hardness change categories as introduced in Sect. 5.2.2, including tendency, magnitude, stability and potential stochastic effects. This will also clarify the separation of significant and insignificant changes. A full break down of the *pathological* cases with respect to our coding scheme (see Sect. 5.2.2) can be found in Fig. 11, “Appendix”. Almost 50% of the tests do not change in hardness significantly (optimal cases). The branch with the changes towards harder reflect our *expected* cases. The branch with the changes towards easier reflect our *pathological* cases. At least 2.82% of the tests are likely to be truly pathological: Significantly easier (more than 50%) in the super-module (therefore harder in the sub), and clear cut stability, i.e. cases where all three measurements in the sub-module are easier than all three measurements in the super-module.

**Fig. 10:**
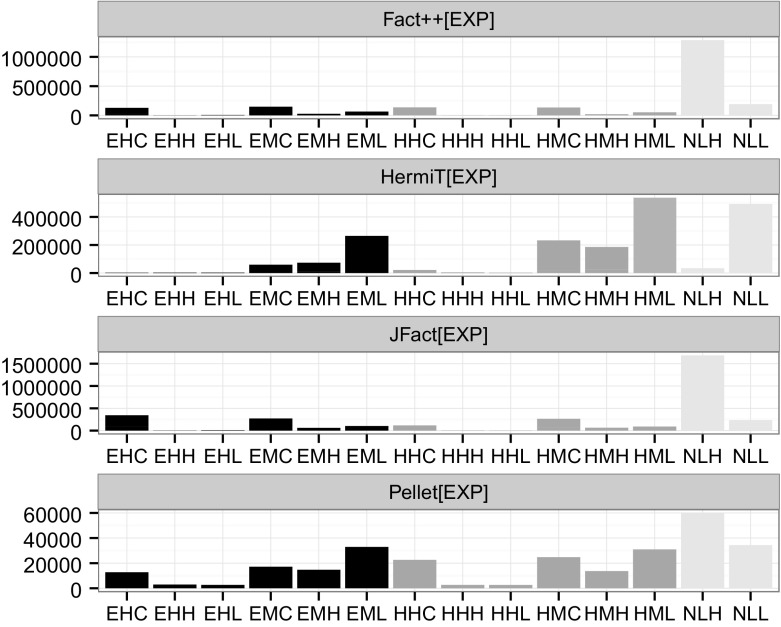
Hardness changes by reasoner: SST. Bin labels x-axis: 1st letter: tendency (easier, neutral, harder), 2nd: magnitude (low, medium, high), 3rd: stability: (clearcut, high, low). Y-axis: number of comparisons. The EHC and EHH categories correspond to our *pathological cases*, the HHC and HHH cases to the *expected cases* and the NLH and NLL to the category of *optimal cases*. The remaining categories are, due to the low number of runs and high variation, neither clearly pathological nor expected. [EXP] indicates that reasoners have been modified to record subsumption tests for the purpose of this experiment

**Fig. 11:**
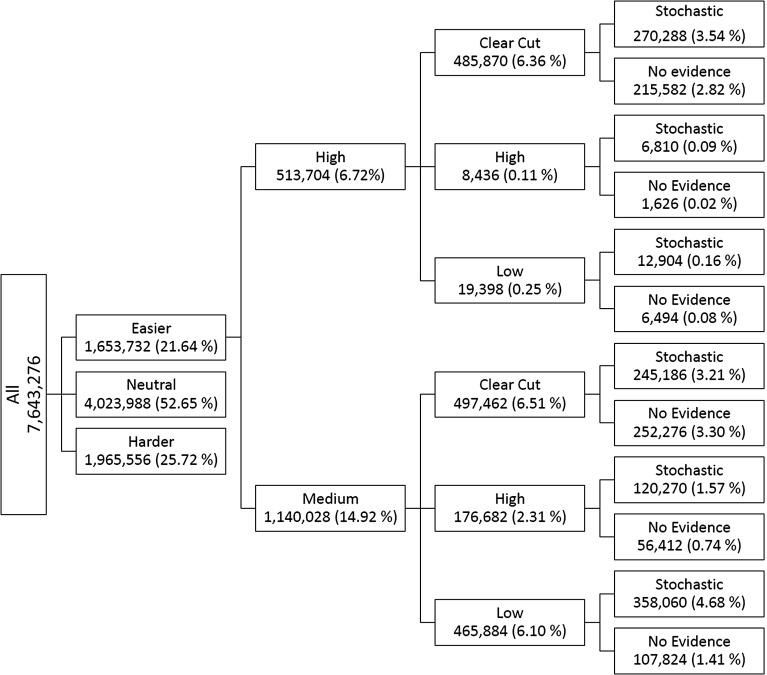
Breakdown of hardness changes for the subsumption tests in the data set that were easier in the super-module (first branch). The second branching reflects the magnitude of the change, the third the stability of the measurement. The number of tests in the “stochastic” category reflects the number of tests for which there was some evidence that the entire classification process (the test was part of) was subject to stochastic effects. Percentages are with respect to overall number of cases

Figure 10 presents the summary of the binning by hardness change category for all four reasoners. FaCT++ exhibits mostly neutral hardness changes, i.e. by far the majority of the tests triggered by FaCT++ do not change by more than 5%. This appears to contradict the observation that Pellet and JFact measurements vary a lot across almost all potential categories. The *pathological* cases as described in Sect. 4.2, corresponding to the EHC category and EHH (tests were much (H) easier (E) with high stability or even clear cut (H/C)), occur rarely (compare also the detailed breakdown in Fig. 11, “Appendix”). Out of the 1,653,732 tests that became easier overall, only 513,704 are of a high magnitude. The bins HHC and HHH correspond the category of *expected* behaviour. We can see in Fig. 10 again that this behaviour is comparatively rare. *Optimal* behaviour is reflected in the NLH category and to a lesser extent by the NLL category, which involve hardness changes that are on average neutral but have a high standard deviation.^24^ These categories are the *by far* dominant category, compare also the neutral (N) changes in Fig. 11 (52.65%, “Appendix”). Interestingly, there appears to be no correlation at all between the difference in the size of the super- and sub-module to the change in hardness (Pearson coefficient less than 0.01).

### Discussion

Our experiments show that on average, reasoners behave in a modularity-sensitive way in terms of subsumption test hardness, i.e. they are able to ignore irrelevant axioms, therefore easifying tests. The magnitude however is, depending on the reasoner, surprisingly low. For Pellet and HermiT at least, pathological changes in subsumption test hardness are often cancelled out by expected positive changes in hardness. This observation suggests that picking a single subsumption test and tracing its hardness through sub- and super-modules in isolation may be misleading. The majority of tests do not change at all under modularity (more than 50%). The positive results for FaCT++ and (to a lesser extent) JFact (easyfication by a factor of 6.18 and 0.42 respectively) are due to a comparatively small number of tests that have extreme changes in hardness, which might be indications for bugs in the implementation, for example during absorption. It remains unclear whether the small number of significantly pathological tests (2.82%), are really pathological, i.e. the consequence of a bug in the implementation, or whether their hardness merely shifted to another test. This can be triggered for example by a changed traversal order due to the difference in signature between the two modules, or random effects in the classification process that we have not isolated. The current experimental setup merely attempts to determine a tendency, i.e. *whether* reasoners are sensitive to axioms that are irrelevant to a particular set of entailments (RQ2).

#### Methodological Reflection

Our original goal was to be able to determine the hardness change between particular subsumption tests $ST(A,B,\mathcal {M}_i,\mathcal {R},\cdot ))$ from $\mathcal {M}_i$ to $\mathcal {M}_2\subset \mathcal {M}_i$. We learned that some tests get significantly harder, most tests do not change in hardness, but some tests also get significantly easier. We therefore conclude that it is insufficient to trace a particular set of tests—the whole population of tests have to be studied at once. We believe that the current experimental design allows for identifying a tendency, but any conclusions about the magnitude of the overall effect should be avoided. First, we ignore tests that are triggered in only one of the two modules entirely. It is possible that the changes in hardness shift to tests outside of the range of tests we are observing. Second, we cannot conclusively isolate stochastic effects if we do not observe the internals of any given subsumption test, and consider changing test orders. It remains to be seen what causes the changes in hardness exactly. Lastly, the sample of ontologies was not meant to be representative of the population, but deliberately biased towards cases that are relevant reasoning in ontologies using expressive languages. A representative sample would take by far too long to be processed with the current analytic pipeline.

As a side observation, we noted the high average variation between individual test measurements across runs. This is most likely a consequence both of stochastic effects and measurement error, due to the lack of accuracy of our timing methods. For any experiments that cannot be statistically significant because of time constraints, this means that the threshold for effects related to individual subsumption tests should be set higher than 5% (we used either 50%, or clear cut).

## Conclusions and Future Work

In this paper, we have established the following main results.

- The impact of tableau subsumption testing on overall classification time is significant only for a *small number of ontologies*, which threatens the applicability of subsumption test hardness optimisation techniques *in general* (not only modular). No conclusions can be drawn on the applicability of optimisation techniques for ontologies outside the performance bounds of our work (i.e., $time(C(\mathcal {O},\mathcal {R},i)) \ge 1\,h$).
- Overall, subsumption test hardness decreased under modularity (by a factor of two in our particular sample). However, the majority (more than 50%) of the tests in our sample did not change at all or only to a very small degree in hardness between the sub- and the super-module; the average decrease in hardness is therefore attributable to outliers.
- The threat of significantly “pathological” cases when considering reasoning with random subsets of ontologies [12] does not extent to reasoning with modules.

Apart from our main conclusions, we made a number of observations that should be interesting to the reasoning community:

- We re-confirm the almost 20 years old results by Horrocks [17] that subsumption tests are generally rather easy. In this work, we characterised subsumption test hardness in great detail.
- Two thirds of the ontology-reasoner pairs in our sample show strong evidence for random effects in the classification process (varying test counts across runs and absence and presence of individual tests). This can cause the hardness of one test to simply “shift” to another, which precludes observation of changes in hardness for individual subsumption tests (rather than population level).
- HermiT and Pellet appear to be sensitive to modularly irrelevant axioms only to some small degree, while JFact and FaCT++ are more sensitive to modularly irrelevant axioms. This suggests that the extent of beneficial effects from modular techniques depends significantly on the particular reasoner.

Datasets and other resources related to the paper can be obtained from http://owl.cs.manchester.ac.uk/publications/supporting-material/phd-matentzoglu/.

Our work is part of a larger agenda to establish the viability of using locality-based modules for optimising reasoning and classification in particular. Despite the relatively modest gains in performance when considering subsumption test hardness reduction all by itself, there is still unexplored potential of modules, such as search space reductions (less tests), or integrating efficient delegate reasoners. We are currently finalising our work on how modularity effects the subsumption test search space and on how we can divide the ontology into a set of, potentially overlapping, modules, to reap the best possible effect from modularity regarding classification time reductions. In particular, we are interested in the potential of modularity-based divide-and-conquer approaches: cutting the ontology into smaller parts and dealing with all the parts separately.

## Figures and Tables

**Fig. 12:**
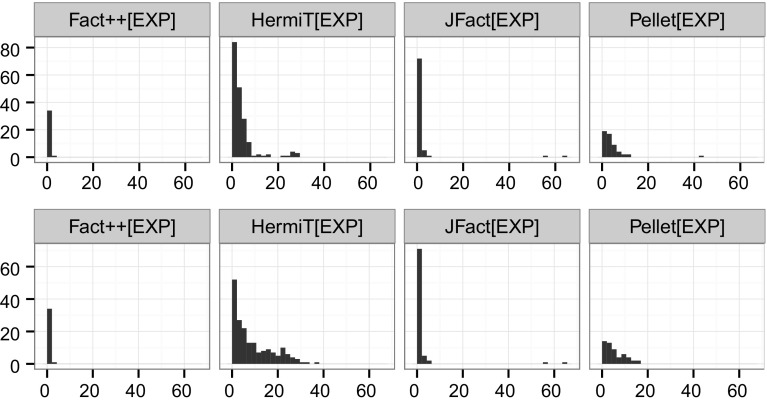
Histogram of COV, by reasoner. *Top* OCT, *bottom* SST

**Fig. 13:**
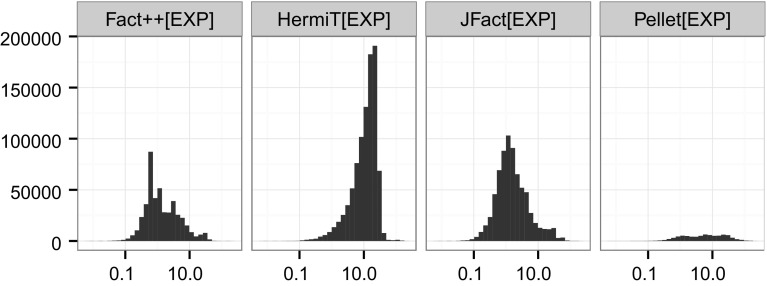
Histogram of COV of subsumption test measurements by reasoner (x:COV, log-scale)

## Appendix

### Full Break Down of Hardness Changes

See Fig. 11.

### Variation of Classification Time Across Runs

See Fig. 12.

### Variation of Subsumption Test Times Across Runs

See Fig. 13.

## Footnotes

1 For brevity, we omit the “hyper” prefix.

2 We use the notion of “run” to denote the execution of a single experiment, for example the classification of some ontology by a reasoner at a particular point in time.

3 For example Targeted Communication [27] for Distributed Description Logics.

4 An aggregation function is typically one of mean, median, maximum or minimum.

5 Note that $\mathcal {O}$ (minus tautologies) is a module of itself.

6 We consider the model size $|\mathcal {I}|$ simply to be the size of the domain $|\varDelta ^{\mathcal {I}}|$.

7 While normally adding non-redundant axioms shrinks the number of models of a theory, in this case we are also expanding the signature.

8 For example Pellet and its internal $\mathcal {EL}$-classifier.

9 Note that this heuristic is not necessarily a bad one: it is very cheap, easy to implement, intuitive and likely to get the right option in lots of cases.

10 For example, stochastic effects induced by the environment (measurement error) or stochastic effects induced by classification non-determinism (variable test order).

11 Concretely: everything that happens from reasoner creation to finishing the classification using the OWL API.

12 This classification is somewhat arbitrary, but it helps to isolate cases for which modular techniques for tableau test avoidance and hardness reduction are inapplicable.

13 This has never been formally verified. Indications of usage are: default shipping with Protégé, citation counts of system description papers and download counts.

14 For technical reasons, the process may have been terminated some minutes after the timeout.

15 For more details on the violations, see [23].

16 For ease of notation, we assume that all runs are indexed with some $i\in \{ 1,2,3 \}$.

17 For example, 75 pure DL ontologies were classified by all four reasoners (*all successful*) but only 23 of these triggered a subsumption test.

18 The reasons for this are still unknown, and investigating them is part of future work.

19 It is important to remember that this excludes the initial consistency check, see Sect. 4.2.

20 See Table 2: 47 ontologies for which all reasoners triggered a test +41 ontologies that all reasoners dealt with but only some reasoners triggered a test + 48 ontologies that not all reasoners dealt with, and at least one of these triggered a test.

21 There were 154 profiled ontologies in total, and 10 out of these caused reasoners to trigger subsumption tests.

22 To date, there is no explanation for this phenomenon.

23 Again: we could have the same number of tests across runs, say 10, but in one run *i*, $ST(A,B,\mathcal {O},\mathcal {R},i)$ is replaced by $ST(A,C,\mathcal {O},\mathcal {R},i)$.

24 The fact that HermiT alone has mostly unstable tests among the neutral cases cannot (currently) be explained.
